# Bio-inspired Analysis of Deep Learning on Not-So-Big Data Using Data-Prototypes

**DOI:** 10.3389/fncom.2018.00100

**Published:** 2019-01-09

**Authors:** Thalita F. Drumond, Thierry Viéville, Frédéric Alexandre

**Affiliations:** Mnemosyne Team, INRIA Bordeaux, Talence, France

**Keywords:** deep learning, not so big data, data prototypes, interpretability, transfer learning

## Abstract

Deep artificial neural networks are feed-forward architectures capable of very impressive performances in diverse domains. Indeed stacking multiple layers allows a hierarchical composition of local functions, providing efficient compact mappings. Compared to the brain, however, such architectures are closer to a single pipeline and require huge amounts of data, while concrete cases for either human or machine learning systems are often restricted to not-so-big data sets. Furthermore, interpretability of the obtained results is a key issue: since deep learning applications are increasingly present in society, it is important that the underlying processes be accessible and understandable to every one. In order to target these challenges, in this contribution we analyze how considering prototypes in a rather generalized sense (with respect to the state of the art) allows to reasonably work with small data sets while providing an interpretable view of the obtained results. Some mathematical interpretation of this proposal is discussed. Sensitivity to hyperparameters is a key issue for reproducible deep learning results, and is carefully considered in our methodology. Performances and limitations of the proposed setup are explored in details, under different hyperparameter sets, in an analogous way as biological experiments are conducted. We obtain a rather simple architecture, easy to explain, and which allows, combined with a standard method, to target both performances and interpretability.

## 1. Introduction

Deep neural networks have had a great success in many domains, especially in visual recognition tasks. From AlexNet in 2012 (Krizhevsky et al., 2012) to Residual Networks in 2015 (He et al., 2016), this class of models has shown outstanding performances at the Large Scale Image Recognition Challenge (aka ImageNet), motivating the use of deep learning in multiple domains such as speech recognition and language processing (LeCun et al., 2015). The key idea is that, at least for threshold units with positive weights, reducing the number of layers induces an exponential complexity increase for the same input/output function (Håstad and Goldmann, 1991). On the reverse, it is a reasonable assumption, numerically verified, that increasing the number of layers yields an input/output function compact representation^1^, as a hierarchical composition of local functions (Goodfellow et al., 2016c).

### 1.1. The Data Requirement Challenge

However, deep learning remains very inefficient concerning data requirements. Most successful results are reported on large databases. For the simple handwritten digit recognition task, on the MNIST database we were already at 60 K images for 10 classes (LeCun et al., 1998). For the complex ImageNet challenge, we reached more than 1M images for 1 K classes (Krizhevsky et al., 2012). While large tech companies may have access to large amounts of labeled and unlabeled data, this is not the case in many domains, where the ability to generalize from only a few tenths of examples per class is required. This limitation not only concerns industrial application requirements but is also a strong limitation as far as modeling cognitive processes is concerned.

Elaborating over this necessity, Mouret (2016) argues for the three basic precepts to deal with small corpus of data, also called micro-data learning: (i) actively search about which is the most relevant data to consider (active learning), (ii) exploit every bit of information (detailed learning), (iii) use as much as possible prior knowledge. In fact, the quick learning ability of humans and animals is largely due to our prior knowledge about the world we interact with and is referred to as transfer learning (Weiss et al., 2016). This can be performed considering general knowledge, beyond *ad-hoc* application dependent choices. In practice, learning from limited data often requires embedding of prior knowledge at some stage, be it at the input data pre-processing, the architecture choice, cost functions, and regularization rules, or even as smart training strategies. Following this track are works focused in few-shot learning (Dong et al., 2017; Rahman et al., 2017), which deals with very few learning samples (usually less than ten), when not only one sample, or zero (i.e., only a priori information).

We would like to study how to investigate a middle range of a few tenths of samples. We may call this situation “not-so-big-data.” The rational for this is two-fold: On one hand it appears that learning entirely new concepts of forms in human (e.g., reading characters; Leroy, 1967) does not require one or a very few number of samples, but more than 10. On the other hand, several industrial applications are able to provide ten to hundred samples for a given perceptual tasks, but not thousands. Such not-so-big-data learning is an extension of few-shot and micro-data learning, reusing several key features such as transfer learning, as reviewed in the section 2.2.

### 1.2. The Interpretability Issue

A step further, we argue that in order to be able to introduce prior knowledge at a general level (and not only using an *ad-hoc* mechanism limited to a single application), the lever is to provide an interpretable processing. Interpretable mechanisms can help to better tune the architecture with small data set, as discussed in this paper. Interpretability also requires a data description easy to explain to a lay audience, and means to provide an explanation for algorithmic decisions that may affect citizen life. We have such requirement in mind in this contribution, as already addressed in Drumond et al. (2017b).

When considering such an issue in the present literature (e.g., Kim et al., 2016, or Zhao and Park, 2016) it appears that interpretability is understood as “results are interpretable.” However, if we really want to propose some easy-to-share and explainable mechanism, in order to be able to state that the process is transparent, we must not only look for interpretable results. We also must attempt to consider easily understood processes, i.e., interpretable mechanisms. This is the reason why in this paper we are going to take the risk to study to which extent a “non trivial but easy to explain” mechanism, used with a small amount of data, can still provide performances close to the best state of the art performances.

### 1.3. On Network Architecture

One of the intuitions behind the success of deep learning is that stacking multiple layers induces learning of a hierarchical representation, a successive composition of local functions that ends up describing higher level concepts as reviewed (e.g., Bengio and LeCun, 2007). This includes, for convolutional neural networks (CNN) with weight sharing, the capability to take global, e.g., translational invariant, features into account (Goodfellow et al., 2016b). That aspect of deep architectures has a strong parallel with our current knowledge of representations in the brain, especially in the visual pathway from retina until the infero-temporal cortex, where object identification is performed. This feed-forward pathway is indeed hierarchical and is responsible for our primary (first ≈100*ms*) visual identification capabilities (Fabre-Thorpe et al., 1998). When considering, however, the complete visual system, a simple feed-forward pipeline cannot represent all the computations taking place in the brain. Identification is only part of our visual system, namely the What ventral pathway, associated to the Where-How dorsal pathway (Milner and Goodale, 1995). Furthermore, there are dynamic mechanisms, feedback modulations, shortcuts, lateral inhibition, to cite a few, that are often disregarded (Medathati et al., 2016).

Concerning the connectivity patterns within the visual system (Bullier, 2001), the capability to mix features from different levels of the hierarchy, as observed regarding the role of the pulvinar (Cortes, 2012), has to be considered. Integrating focus of attention within certain regions of the feature space (Saalmann et al., 2012), and other functionalities such as on-the-fly adaptation to incoming data, in link with the few-short learning paradigm (further discussed in section 2.2) is also important to take into account^2^. All these functionalities require rather general recurrent architectures (Medathati et al., 2016). The possibility to provide a framework in which not only feed-forward or specific recurrent architectures can be taken into account is addressed by other works (Viéville et al., 2017) and is beyond the scope of this paper. The present proposal is mainly inspired on how the infero-temporal cortex has a kind of prototypical representation, with neurons tuned to respond to particular categories (Viéville and Crahay, 2004).

### 1.4. Hyperparameter Dependence

Automated machine learning^3^ is “the process of automating the end-to-end process of machine learning,” because the complexity of adjusting the hyperparameters, including selecting a suitable method, becomes easily time-consuming when not intractable. To this end, the general idea is to consider a standard machine-learning algorithm and to add on “top of it” another algorithmic mechanism, e.g., another machine learning algorithm dedicated to automatic hyperparameter adjustment, with the caveat of generating other hyperparameters for the meta-learning algorithm and without formal guaranty that this accumulation of mechanisms is optimal.

What corresponds to hyperparameters in the brain or for a fully autonomous system is managed in three ways: It is either modulated by other structures (such as the different cortico-thalamic pathways through the basal ganglia; McHaffie et al., 2005) and at different time-scales (such as short-term versus long-term synaptic plasticity; Cooper et al., 2004), or integrated as learning parameters, the system being able to learn new parameters and to adapt also the way it learns as a coherent process. A third track is to design robust processes in the sense that hyperparameters precise values are not critical. A key issue in a distributed system such as the brain, is that hyperparameters are usually global while still implemented via local processes (e.g., neuro-modulation). Some are also related to the network construction (e.g., the number of layers).

Here we are going to carefully study these aspects, in order to make explicit to which extents the proposed method is related to robust hyperparameters, thus without requirement about questionable value adjustment. If not, the hyperparameter adjustment is going to be managed using standard hyperparameter adjustment methods, and the interpretability of the such meta-parameter adjustment is also going to be made explicit.

### 1.5. The Present Contribution

Bio-inspired improvements of deep learning extend far beyond the three issues reviewed here, as discussed recently in Marcus (2018). The biological plausibility of deep-learning is another issue, see Bengio et al. (2016) for a recent discussion. In a nutshell, there is a clear correlation between deep-learning CNN states and the primary visual cortex macroscopic activity as observed in Mensch et al. (2017), while the contribution of deep-learning as a tool to better understand the brain is not obvious as explained in Medathati et al. (2016). Nevertheless, these issues are beyond the scope of this paper.

Here we would like to revisit the notion of prototypes, as a tool to improve deep learning on not-so-big data, considering some aspects of the brain architecture and addressing also the hyperparameter dependence issue. There are multiple definitions and usages of what is called prototypes. Here prototypes are centers of a k-means procedure over training samples, acting as data-representatives in the feature space. The present work explores this notion of prototypes for interpretability and classification under not-so-big datasets, building up on the idea introduced in Drumond et al. (2017b) with more comprehensive experiments on real datasets and including comparison with few-shot meta-learning methodologies. This work focuses on convolutional networks and applications to vision, although what is proposed in this paper is general and applicable to other deep learning frameworks.

Our main claim is that such data prototypes may help address the not-so-big data issue in an interpretable fashion, considering a few bio-inspired aspects made explicit in this introduction. Proposing a solution to both aforementioned issues—“not-so-big-data” learning together with data and process interpretability—is the main contribution of the paper. The key point of our proposal is the notion of data prototypes, as made explicit in the sequel. In order to introduce this notion, we need to discuss one aspect of network architecture and point out issues regarding the hyperparameters.

In the next section we are going to review the literature related to this subject, then formalize the model description, in order to experiment the idea in the subsequent section, allowing us to discuss based on these results how the issues quoted here can be addressed, while proposing several perspectives of this work.

## 2. Related Works

### 2.1. Prototypes in Literature

The term prototypes can be found in the literature under different meanings: a priori information, representation in clusters, quantification of space, as we discuss now.

Jetley and Torr (2015) proposes a prototypical priors layer, with a priori chosen prototype images of road signs, encoded using a HoG (histogram of oriented gradients) descriptor, and added as fixed units of the penultimate layer. Here, the network is ultimately trained to match the HoG representation of the prototypes but it is assumed to exist a standard representative image per class in the input space to be encoded as a prototype unit.

In prototypical networks (Snell et al., 2017), prototypes are defined as class centroids in the feature space spanned by the embedding CNN. In this simple approach, the nearest-prototype is used to classify a given sample. This proved to be effective on the considered datasets. However, this does not respond to the scenario of metric learning proposed in Song et al. (2017). Also derived from this work, Gaussian prototypical networks (Fort, 2017) predict a covariance radius for each prototype, yielding some insight on the discriminating force of each of them.

Self-organizing maps, or the dictionary sparse representation methods (Rubinstein et al., 2010), perform a prototypical sampling on the data space (Hecht and Gepperth, 2016) and allow to represent what is known about a data distribution, using methods quoted in prototypical networks and known as optimizing statistical criteria (Banerjee et al., 2005).

Bio-inspired models also consider the notion of prototypes, as in e.g., (Serre et al., 2007) which introduce a general framework for the recognition of complex visual scenes, that follows the organization of visual cortex building an increasingly complex and invariant feature representation and considering a redundant dictionary of features for object categorization. Furthermore, Viéville and Crahay (2004) has related the notion of prototypes to a SVM model of the inferior temporal object recognition brain area.

### 2.2. Few-Shot Learning and Learning to Learn

The importance of learning new concepts from small data-sets motivated the study of few-shot learning tasks. Few-shot learning refers to the ability of learning to discriminate between *N* unseen classes given *k* examples of each, with *k* usually below 5 (Triantafillou et al., 2017). This task is also referred to as k-shot N-way learning. One-shot learning is a special case of this setting, where the system must generalize from a single example for each class. Slightly different is zero-shot learning, where no example of a given class is available in the target domain (e.g., images) whereas information on the categories originates from other domains (e.g., textual descriptions) (Goodfellow et al., 2016d).

Learning a deep discriminative model for a large number of classes has high data requirement, and is prone to overfitting if applied directly in a few-shot data framework. For this reason, usually some form of transfer learning is used to tackle this problem: a model is trained on other classes before attacking the *N* new ones. Since the model learned in this pre-training phase will have to be adapted to the target classes, few-shot classification can also be seen as a form of “learning how to learn,” which is a very sensible framework when the goal is to learn whichever new categories to come. In this spirit, pre-training is a meta-training phase, where we learn a meta-model that can learn any set of *N* classes given to it. Using the model on new k-shot N-way tasks corresponds to a meta-testing phase, where for each task some inner training may take place.

There are different ways of implementing this meta-learning setting^4^, but any such should have a meta-training and meta-testing phase, with their respective class-wise disjoint datasets *D*_*Mtrain*_ and *D*_*Mtest*_. Details on each phase vary between propositions, but globally meta-training works like some sort of pre-training, where the model is trained on some discriminative task over the classes in *D*_*Mtrain*_, while constructing a representation that will be useful to generalize to new classes in *D*_*Mtest*_. In this sense, this methodology can still be seen as a form of transfer learning. Later, meta-testing phase will consist of actually performing the k-shot N-way task multiple times, for different subsets of *N* classes drawn from *D*_*Mtest*_. Each subset itself has to be divided into training and test splits, in order to have *kN* reference samples for the new classes from one and evaluate performance on the other.

To give a clearer example, let us present a common organization of meta-learning: episodic training. First proposed by Vinyals et al. (2016), it has continued to be adopted in other recent works (Santoro et al., 2016; Fort, 2017; Ravi and Larochelle, 2017; Ren et al., 2017; Snell et al., 2017). Arguing that approximating meta-training to meta-testing conditions could enhance learning, they proposed that, during each step in the meta-training phase, the model be trained on a different k-shot N-way task, with new *N* classes drawn from *D*_*Mtrain*_. Each such task is called an episode, and demands not only *kN* examples as a training (or support) set but also some examples of the *N* classes to serve as a test or query set. During this phase, the error over this query set can be used to adjust the model, while in meta-testing only the support set will be used as a reference to classify the query samples.

In line with the above discussion, many of the recent works focused on this problem, seek to learn an embedding space over the meta-training set that will hopefully generalize for unseen classes in meta-testing. This common feature space allows to compare test with training examples to decide on their class, usually with some kind of nearest neighbors algorithm, but sometimes resorting to more complicated models. Siamese nets (Koch et al., 2015) are an early example of such models, in which two identical networks are used to map a pair of examples into a learned metric space so that they can be compared via a distance function. The whole network is trained to predict whether an input pair belongs or not to the same class, this being repeated for multiple pairs. Matching networks (Vinyals et al., 2016) work on a one-shot setting, also relying on learning a network that will map support examples of each class to an embedding space, to be later matched to the query example. The embedding is composed by CNNs providing inputs to LSTMs, providing a context aware embedding that models dependence between the CNN feature vectors for each support point, and also between support points and query point. Prototypical nets (Snell et al., 2017) also learn an embedding based on a CNN, with the matching between support and query samples performed by a nearest class means classifier. The CNN is adjusted during meta-training, with a cross entropy loss function over the points in the query set, for multiple episodes.

This line of work is in close relation with metric learning, which aims to adapt a metric function over feature vectors for a given dataset (Bellet et al., 2013). If a significant metric is learned, distance-based classification becomes a relevant alternative, as exemplified by Mensink et al. (2013). They consider metric learning methods for two distance-based classifiers, the k-nearest neighbor (k-NN) and nearest class mean (NCM), and propose new methods for NCM allowing to model more complex class distributions with multiples centers per class. This possible complexity in class distribution cannot be captured by local metric learning methods, as is the case with current deep metric learning. As discussed by Song et al. (2017) they are incapable of identifying scattered classes, with multiple clusters in the space. In response, they propose to learn an embedding function that directly maximizes a clustering metric (normalized mutual information).

Using memory augmented networks has also been explored in the context of few-shot learning (Santoro et al., 2016). The idea is to build on top of a Neural Turing Machine (Graves et al., 2014), an implementation of a content-based access memory for neural networks, adapting it to the one-shot learning task. This is yet another meta-learning approach, where the recurrent network is not trained to predict directly a specific set of classes, but tries to predict the right classification at each time-step based on the sample-class associations it could learn and keep in memory on the previous time steps. It still needs to see a large number (more than 10 k) of episodes to make good predictions, incrementally making better predictions as it sees up to 10 examples of the same class. Compared to human memory, its functioning could be seen as a working memory, that can match new samples to recently seen examples but limited to a low amount of distinct categories.

### 2.3. Interpretability

Interest in the field of interpretable machine learning has raised in the last years, partly inspired by the ever-growing impact of machine learning systems in society. Nevertheless, interpretability is a broad term still lacking a precise definition, with open discussions on what it is and how to quantify it (Lipton, 2016; Doshi-Velez and Kim, 2017). One common understanding is to equate it to explainability—the ability to provide explanations to a model's predictions or, even better, the reasoning process behind its predictions.

Understanding the reasoning behind complex models such as deep neural networks is a difficult task. In visual recognition applications, one common effort is to try to visualize what types of features have been learned by certain units or layers of a convolutional network. This is usually achieved by optimizing network input to maximize the activations of the layer of interest. Since the first visualizations produced with DeconvNet (Zeiler and Fergus, 2014), there have been many propositions to improve the quality of the input reconstruction, including the use of regularization priors that enforce more “natural-looking” images (see Olah et al., 2017, for a review). Another way of producing such images is to search the training set for image patches maximizing the activations. This approach has the advantage of producing real examples, although not necessarily specifying which features in the image led to it to be put in a particular category.

In-between these feature visualization strategies is the problem of attribution, where the goal is to identify which regions of the input image were responsible for maximizing a chosen activation (Bach et al., 2015; Sundararajan et al., 2017). An example of attribution procedure is LIME, a method that locally approximates the model around the output prediction and goes back to the input image highlighting superpixels most responsible for its predicted class (Ribeiro et al., 2016). Other works are interested in generating some salience map information over the input image (Bach et al., 2015; Shrikumar et al., 2017). While feature visualization is still abstract and away from verbal human-level explanation, attribution or salience maps are grounded on real images and can provide some level of justification.

Even though these methods were developed having deep CNNs in mind, some of them can be generally applied to explain other classes of models. The explanation procedure itself can be seen as an explanation model, trained to provide justifications given the inputs and outputs of the black-box prediction model. Lundberg and Lee (2017) defines additive feature attribution methods, a class of local explanation models, unifying diverse approaches from literature (including Bach et al., 2015; Ribeiro et al., 2016; Shrikumar et al., 2017). Another way to use an accessory model as explanation is to distill the network into a class of allegedly interpretable models—such as decision trees—training the explanation model to mimic the networks predictions (Frosst and Hinton, 2017).

## 3. Model Proposed

### 3.1. Model Architecture

Here we introduce a prototype matching layer as proposed in Snell et al. (2017), after the embedding provided by a CNN, defined by a set of prototypes sampling the input distribution in the feature space. This layer is a softmax taking both the sample features and the sample prototype proximity into account. This allows grouping examples with respect to the induced metric learned by the CNN layers, and taking the competition between prototypes into account. Differing from Snell et al. (2017), this output feeds an additional final softmax classifier layer, that correlates the prototype's matching to class labels. This allows to introduce a soft but sparse prototype-to-class label assignment: A given prototype is encouraged to represent a unique class, but in ambiguous situations this mapping is not hardwired. It also differs from alternative mechanisms such as support-vector machines, Kohonen-like maps, or radial basis functions (such as e.g., Lyu and Simoncelli, 2009).

Some specificity with respect to usual architectures using prototypes are taken into account here:

- The prototype layer is not the final layer but an intermediate upper-layer of the architecture, as a complementary information to perform the perceptual task. Here classification is the task, but this extends to other perceptual tasks.
- A prototype is a “data prototype” in the sense that its role is not only to reveal if a neighborhood of the feature space belongs to a given category of the classification task, but also to represent at a macroscopic level the feature space itself (e.g., which regions are to be taken into account, whether a category corresponds to a connected region or not, whether a region contains adversarial samples, etc). As a consequence, we neither hard-wire sample to prototype matching nor prototype to class label assignment, but let the estimation provide the best description of the data, given the classification task, in an interpretable way, as discussed below. This is quite different from the related works reviewed in the previous section.
- Given a dataset, the final task-related layer is fed with two alternative combined inputs: (i) The whole feature sample vector, and: (ii) The data in relation with the prototype space. With the former choice only, the prototype layer role is simply to represent the data in the feature space but is not involved in the classification task. With the latter choice only, the large dimensional feature sample (here of dimension 2,048, see details in the next section) reduces to a very low dimensional space (<50 in our setup). This very likely introduces some bias, but may limit overfitting.

Let us now turn to the formal detailed description of the model.

### 3.2. Model Specification

We consider^5^ a set of labeled images {···(**I**_*i*_, *l*_*i*_)··· }, *i* ∈ {1, *M*}, labels being finite *l*_*i*_ ∈ {1, *N*}. This input space I is embedded in a feature space X of huge dimension *D* through a function *f* : I → X, here defined by the CNN. We introduce the notion of prototype in order to discriminate between twisted features at the output of some CNN.

To this end, we propose to consider the estimation of a fixed-size set of prototypes in the feature space **p**_*j*_, *j* ∈ {1, *J*}, *J* ≥ *N*. The fact this set is fixed is going to be discussed at the experimental stage, showing that the hyperparameter *J* is robust.

We are in a supervised learning paradigm considering samples {···(**x**_*i*_, *l*_*i*_)··· } providing examples of association between features and categories.

We proceed in two steps. First, *q*_*j*_(**x**) = *p*(**x** ∈ *Q*_*j*_) is the probability for the feature vector **x** of a sample, to be associated to the *j*-th prototype. This association is written as **x** ∈ *Q*_*j*_, where *Q*_*j*_ is the set of samples associated to the given prototype. Interestingly enough, our model does not implicitly assume that the *Q*_*j*_ form a partition of the feature space, as discussed in Appendix A5. These regions may overlap or uncover the whole space. Furthermore a sample may be sparsely represented, thanks to the softmax criterion, by several prototypes as in a dictionary representation method (see e.g., Rubinstein et al., 2010). We approximate *q*_*j*_(**x**) as a function of the proximity −||**x** − **p**_*j*_|| to the prototypes, writing:

(1) $$\begin{array}{l} q_{j}\left(\text{x}\right)=\nu_{j}\left(-||\text{x}-{\text{p}}_{j}|{|}^{2}/2\right), \end{array}$$

where ν_*j*_(·) stands for the softmax distribution, the related *D* × *J* weight matrix of this layer corresponding to prototypes coordinates in the feature space X. See Appendix A1 for a detailed presentation of this equation.

Then, *c*_*l*_(**x**) = *p*(*l*|**x**) is the probability for a sample to be associated to the label of index *l*. For the purpose of analyzing different aspects of the given architecture, we consider two alternatives: *direct* use of the prototypes and *combined* use of prototypes and features.

#### 3.2.1. Direct Use of the Prototypes

The label of sample is estimated by another softmax layer:

(2) $$\begin{array}{l} c_{l}\left(\text{x}\right)=\nu_{l}\left({\text{w}}_{l}^{T}\text{q}\left(\text{x}\right)\right), \end{array}$$

the related *J* × *N* weight matrix corresponding to the prototype-to-class association. This design choice introduces an important dimensional reduction (see e.g., Shalev-Shwartz and Ben-David, 2014, Chapter 23) in the processing. It is a strong choice and it is going to be numerically studied against a vanilla alternative made explicit in the next subsection.

Considering an approximate cross-entropy criterion to adjust the parameters given sample features **x**_*i*_ and their label *l*_*i*_ yields the following minimization:

(3) $$\begin{array}{l} C=-\underset{X}{\int }p\left(\text{x}\right)\log\left(c\left(\text{x}\right)\right)+\frac{1}{C}|{\text{w}}_{l}{|}_{d}≃\\ -\frac{1}{M}\sum_{i}\log\left(c_{l_{i}}\left({\text{x}}_{i}\right)\right)+\frac{1}{C}|{\text{w}}_{l}{|}_{d}, \end{array}$$

where *d* ∈ {1, 2} corresponds to the L^1^ or L^2^ norm, while *C* is a hyperparameter weighting the regularization term.

A straightforward derivation (see Appendices A2, A3 for the details) of the related normal equations leads to an estimation-minimization method:

- The ∂_**p**_*j*__C = 0 equation yields a k-means estimation of the prototypes given the samples, weighted by the prototype proximity. This deep relation between divergence estimation and k-means algorithms is known (Banerjee et al., 2005) and will allow us to derive an efficient implementation, and to minimize the role of meta-parameters for this step. In our implementation we have experimented that considering only a standard k-means initialized by a k-means++ mechanism is numerically sufficient. - $\partial_{{\text{w}}_{l}}C=\underset{i}{\sum }\left[\delta_{l=l_{i}}-c_{l}\left({\text{x}}_{i}\right)\right]\text{q}\left({\text{x}}_{i}\right)+R$, where R stands for regularization terms, yields a Hebbian-like interpretable 1st order rule for adjusting the prototype-to-class association, as a function of each training sample a priori class label.

#### 3.2.2. Combining Prototype-Encoded and Original Features

The combined model uses the probabilities for a given input sample to correspond to the prototypes in order to predict the class. The input sample is encoded via these numbers. It is obvious to decode such information, i.e., to reconstruct the predicted sample given this information:

(4) $$\begin{array}{l} \tilde{\text{x}}\overset{\text{def}}{=}\underset{j}{\sum }q_{j}\left(\text{x}\right){\text{p}}_{j}, \end{array}$$

where $\tilde{\text{x}}$ is the simple Bayesian estimation of **x** given *p*(**x** ∈ *Q*_*j*_), providing^6^ a simplified form of linear autoencoder mechanism (Goodfellow et al., 2016a).

When *D* ≫ *J*, it might be the case that the prototype information is insufficient to allow a good classification performance. Conversely, using a large *J* may interfere with interpretability, a large number of prototypes being harder to analyses and eventually being less meaningful. To this end, we are also going to also consider

(5) $$\begin{array}{l} c_{l}(\text{x})=\nu_{l}\left({\text{w}}_{l}^{T}(\text{x}\;|\;\tilde{\text{x}})\right)=\nu_{l}({\text{w}}_{l}^{\prime T}\text{x}\;|\;{\tilde{w}}_{l}^{\prime T}\tilde{x}), \end{array}$$

in the upper classification layer, where | is a vector concatenation operator and ${\text{w}}_{l}^{\prime T}=({\text{w}}_{l}^{\prime T}\;|\;{\tilde{w}}_{l}^{\prime T})$. This provides the following layer with both features and prototype-encoded information to make a classification decision. Additionally, comparing ${\text{w}}_{l}^{\prime T}$ with ${\tilde{w}}_{l}^{\prime T}$ allows to numerically evaluate the contributions of both paths identifying to what extent the reduction of information from the whole data features to a prototype-encoded representation is relevant to the classification decision.

#### 3.2.3. Relation Between Prototypes and Category Information

Another issue with respect to the state of the art is, as proposed in Rippel et al. (2016), to consider the prototypes as a mixture between unsupervised information (sampling the feature space without any knowledge) and supervised hardwired information, each prototype being hardwired to a given category. In order to further understand the relationship between the data prototypes and the categorization, since in alternative approaches prototypes are often “hard-wired” to a given category, we have also investigated an extension of the k-means algorithm step on the learning set, taking also the learning sample category into account.

To this end the following extended metric for the k-means algorithm applied on learning samples is considered:

(6) $$\begin{array}{l} d\left(\left({\text{x}}_{i},l_{i}\right),\left({\text{x}}_{i^{\prime }},l_{i^{\prime }}\right)\right)=|{\text{x}}_{i}-{\text{x}}_{i^{\prime }}{|}_{2}+\beta \underset{j}{\sum }\delta_{l_{i}\neq l_{i^{\prime }}} \end{array}$$

In words we augment the feature space by new dimensions corresponding to each categories in order to move apart samples related to different categories. Clearly for β = 0 this reduces to our original setup, whereas for large values of β, we make explicit in Appendix A4 how this allows to hard-wire prototypes to categories.

This complementary tool is not related to performances but to only better understand this aspect of the problem. Additionally, it is important to understand that there is a deep relationship between a softmax classification layer and a prototype representation, as discussed in details in Appendix A7.

## 4. Experiments and Results

Here we perform a series of experiments to gain intuition about the proposed model, test its performance under a few-shot setting and demonstrate its potentialities as an interpretable alternative. In summary we used two experimental methodologies: first a k-means based implementation over fixed features, followed by a second end-to-end implementation used in a meta-learning setting. In both cases, we work under few-shot learning conditions, exploring the performance of these models for not-so-big data situations.

### 4.1. Datasets

A known benchmark dataset for few-shot learning is the Omniglot dataset (Lake et al., 2011). It consists of images of 1,622 characters from 50 different alphabets, each drawn by 20 different people. The small number of samples per class and the large number of classes make it a challenging problem to learn all the classes simultaneously. Therefore we will work on episodes: class subsets of *N* classes randomly picked among the 1,622 characters (see Figure 2: Left for an example).

**Figure 1:**
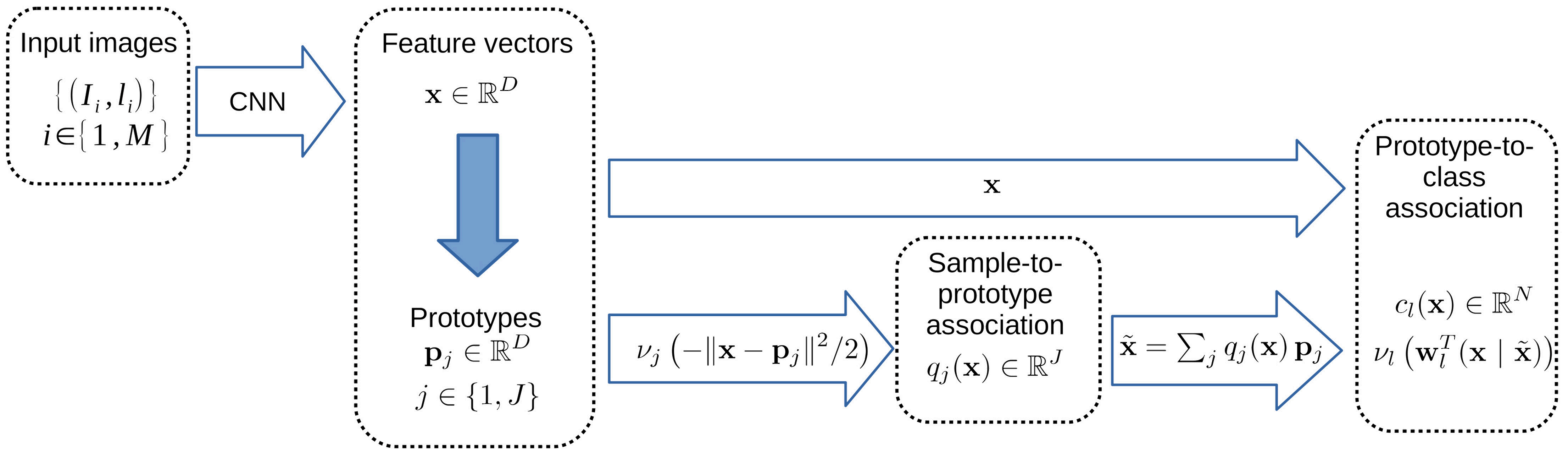
The three layer model. The input data is fed to a convolution neural network, transforming the original image into a set of feature vectors. Then the information in this high dimensional feature space is summarized via a set of prototypes, in order to understand the landscape. Finally, the relation between a given data point and the label to infer is calculated through their proximity to the prototypes.

**Figure 2:**
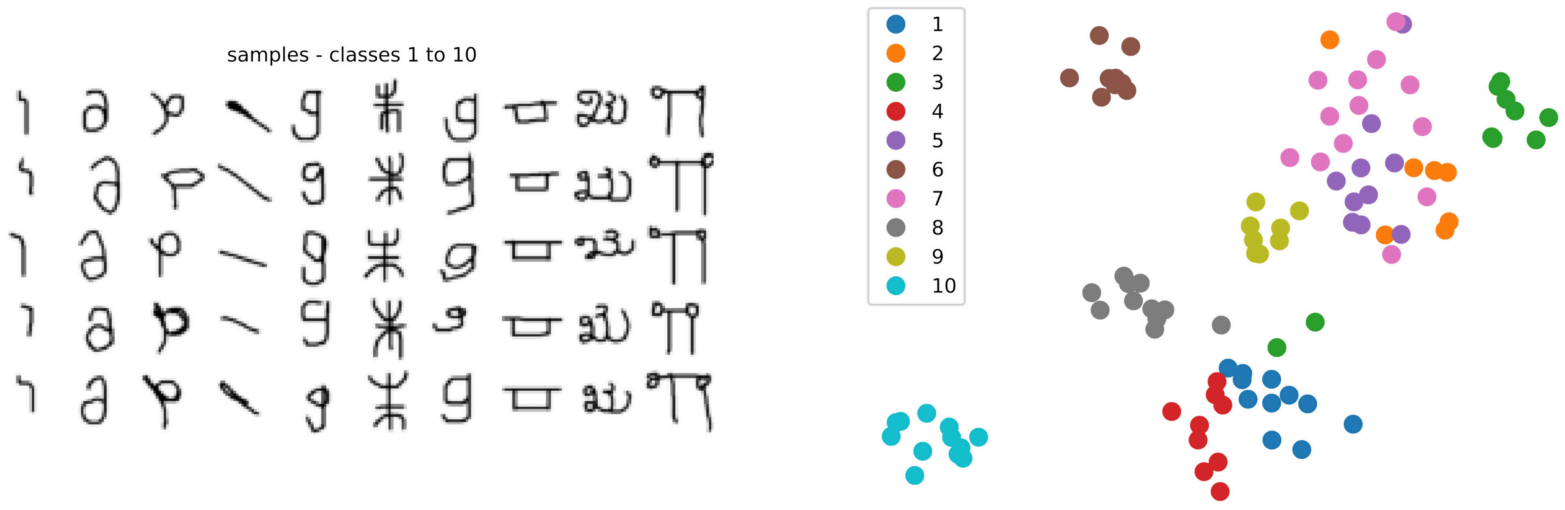
**(Left)** Sub-sample of 10 classes from the Omniglot dataset, showing 5 samples per class (total is 20 per class). **(Right)** T-SNE visualization of the Inception v3 features for the 200 samples of this subset, see text for details.

To study the effect of increasing training set sizes, we will use two larger standard benchmark datasets: MNIST^7^ and CIFAR10^8^. Both datasets comprise 10 different classes. MNIST has 28 × 28 images of handwritten digits, 60 K samples in the training set and 10 K in the test set. CIFAR10 has 60 K 32 × 32 color images of 10 object categories, with 50 K in the training set and the remaining 10 K on the test set. From these datasets, it will be possible to sample training subsets ranging from few-shot to many-shot situations.

### 4.2. Studying the Model Under Fixed Features

Contrary to usual few-shot learning paradigms we do not consider here meta-learning as discussed in section 2.2, but a transfer learning paradigm: by using a general purpose pre-trained CNN, we skip any meta-training routines that allow learning CNN filters on a particular few-shot dataset. We use fixed features extracted with a standard CNN architecture pre-trained on ImageNet data, namely Inception v3 (Szegedy et al., 2015), that has successfully been used in transfer learning settings (for instance Shin et al., 2016; Esteva et al., 2017). Features are normalized in the range [0, 1]. Based on features extracted from the penultimate layer of this network, our model has to learn to predict from very few samples (see Figure 2:Right for a visualization example of this feature space for a sample episode of Omniglot dataset). Fixing the embedding network allows to isolate and assess performance variations due to the final layers of the model, under equal input features. Assessing whether the tested models allow learning of a more appropriate representation is addressed in section 4.4.

Except for experiments with increasing training set sizes (section 4.3), all experiments in this section are performed on Omniglot dataset. In comparison to meta-learning methodologies, we are skipping the meta-training phase, where the embedded representation is learned, and going directly to meta-testing, where for each independent episode, the model is actually only learning to predict from few-shot data, for a certain quantity *N* of classes. Thus we operate direct learning on each episode, with a training data set for parameter estimation and a test set for checking generalization. Each episode is split in half, with 10*N* samples for training and 10*N* samples for testing. When hyperparameter tuning is needed, 5 × 2-fold cross-validation over the training set is used, leaving 5*N* samples to train and 5*N* to validate. Once the hyperparameters are chosen, the model is re-trained on the entire training set. To see how the model performs over the entire dataset, observations are repeated for a large number of episodes.

With those two simplified design choices (using fixed features and performing direct learning), these results cannot be fairly compared to other few-shot meta-learning approaches (this will be addressed in section 4.4). More precisely, we withdraw from learning the CNN layers, which are not adapted to the target data to give a specific representation, relying on transfer learning instead. Moreover, using the direct model only, it drastically restrains the classification number of degrees of freedom from thousands to a few tenths. This is done on purpose in order to study to what extents this compromises the result. Nevertheless, we also know how to also ensure performances, using the so-called combined model. In both cases, we compare our propositions to other classical classifiers tested in the same setting, namely: softmax, SVM, and nearest centroids.

To visualize how data samples and prototypes are distributed in space, we obtain a 2D non-linear projection using T-SNE (Maaten and Hinton, 2008) over the data samples feature vectors together with the prototypes coordinates *p*_*j*_ in the feature space, as shown in Figure 2: Right. Such mechanism preserves local neighborhood of closed samples, whereas long range distances can not be interpreted since skewed by the non-linear projection: The map only reflects the similarities between the high-dimensional inputs in a stochastic, but not metric way.

Code and further analysis for this section and section 4.3 are available at https://gitlab.inria.fr/mnemosyne/data_prototypes.

#### 4.2.1. Study of a Sample Episode

Hyperparameter adjustment is a key factor in the success of any machine learning model. This first analysis focuses on gaining intuition on the behavior of the model on a sample episode with *N* = 10 classes (shown in Figure 2). The aim is to study its hyperparameters, experiment with visualization of prototypes and data samples in a common space and try to understand the relation between prototypes and their attributed class. In this section we consider only the hyperparameters introduced by the formulation presented in section 3.2. More details on these and other hyperparameters can be found in Appendix B.

**Figure 3:**
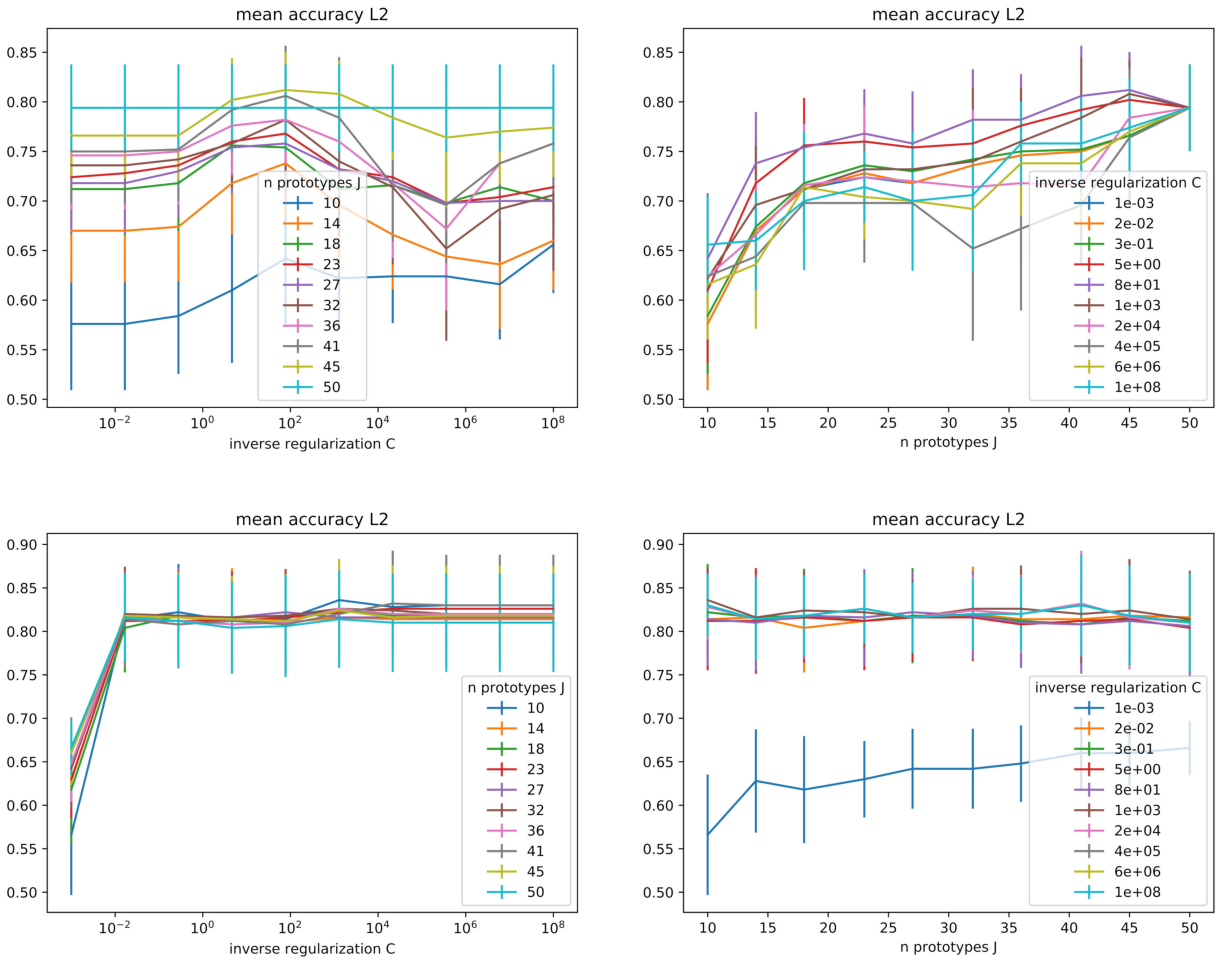
Mean accuracy (with standard deviation bars) obtained during grid search for the inverse regularization parameter C and the number of clusters, under L2 regularization. **(Top)** Direct model. **(Bottom)** Combined model.

In the current experimental setting, our base model has two hyperparameters: the inverse regularization strength (*C*) and the number of clusters, thus of prototypes (*J*). We explored 10 values of *C* ∈ [10^−3^, 10^8^], and 10 values of *J* ∈ [2*N*, 5*N*], where *N* = 10 is the number of classes (see Figure 3). For the direct model under L2 regularization, we can identify that average performance is stable for *C* ≤ 10^−1^, and more sensitive around larger C values in (10^2^, 10^6^], raising around *C* ≤ 10^2^. For L1 larger values are preferred, presenting a stable score as soon as *C* ≤ 10^2^. For the combined model, we have a behavior similar to direct-L1 under both regularizations, with any values above a certain threshold being equivalently good (10^−2^ for L2 and 10^1^ for L1). Since no clear preference could be stated between L1 and L2 regularization, both regularizations keep being analyzed in the following (and are included in the comparative analysis in section 4.2.3).

Regarding the number of prototypes *J*, as visible in Figure 3, a higher number of prototypes is preferred by the direct model, while the combined model does not seem to depend on it. However, for the direct model, using a too large number of prototypes would ultimately amount to a nearest-neighbors-like algorithm and possibly counteract on their interpretability purpose (too many prototypes representing too many base concepts to support a simple explanation), while overfitting and poor generalization performances are expected. With the combined model, we can avoid this issue by choosing a lower number of prototypes while keeping high performances.

**Figure 4:**
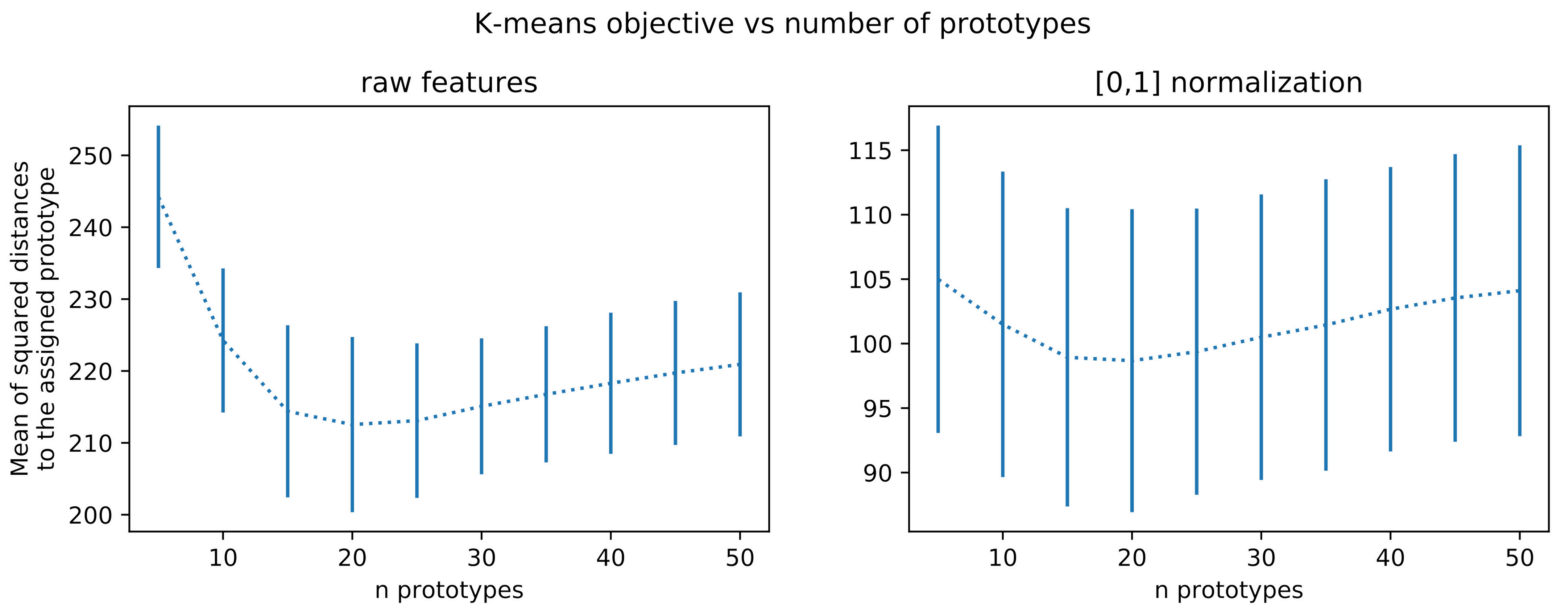
Average training loss on the k-means algorithm as a function of the number of prototypes. The mean square distance between samples and prototypes decreases until a plateau (sometimes called elbow) and then either re-increases, as visible in this example, or do not significantly re-decreases as shown in Figure 9 for other datasets.

Nevertheless, a minimal number of prototypes can be important for interpretability, but can be ignored when looking only at accuracies in cross-validation. This motivates using another method for choosing the appropriate number of prototypes. As far as data representation is concerned, we can simply rely on usual k-means prototype count adjustment methods. This is usually done by sweeping some range of values for the number of clusters and keeping the one minimizing its loss or, in case of saturation as *J* grows, keeping the lowest value that reaches close to minimal loss. We exemplify this procedure in Figure 4 for an Omniglot 10-shot episode, which suggests a number of prototypes around 20. Statistical significance of the observed elbow curve has been verified. More precisely, considering a least-squares adjustment of a parametric elbow model against a constant value model, the related Fisher ratio test probability threshold is *p* = 0.2 on the non-normalized values. It is only *p* = 0.3 on the normalized values, and it is more than *p* = 0.001 (non-normalized) and *p* = 0.2 (normalized) on the MINST data set reported on Figure 9 and only *p* = 0.3 for the CIFAR 10 data set. We have tested for several parametric models (cubic, piece-wise linear, exponential) and obtained the same results. We hypothesize this is due to due to very small set of data used (10 samples by data point). However, as far as interpretability is concerned it is still interesting to have an estimation of a reasonable order of magnitude of the number of prototypes. We also are going to observe in the sequel that this number is not critical regarding performances, so that this rough estimate is sufficient.

In summary, we can choose the number of prototypes in the clustering phase based directly on its unsupervised loss, optimizing the number of prototypes for data representation. Altogether, the key points here are that this hyperparameter is to be considered more as a way to optimize the data interpretability than the classification performances, with the possibility of being automatically adjusted.

**Figure 5:**
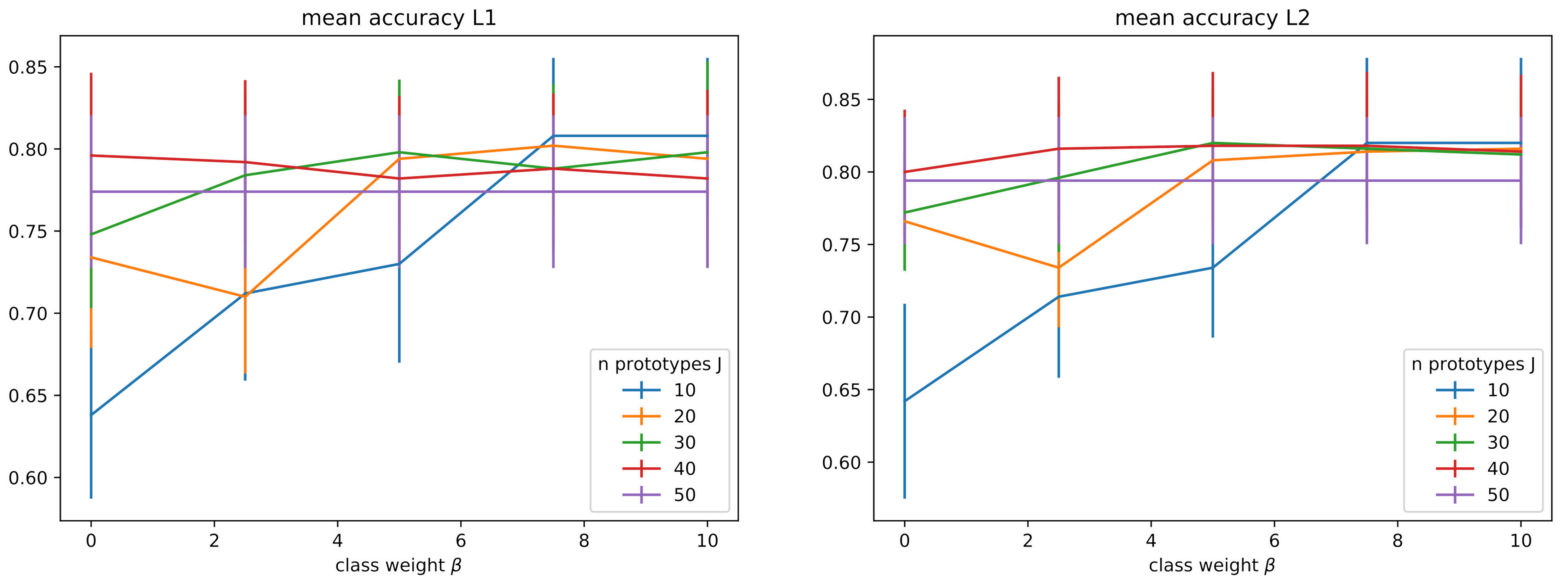
Verification that the introduction of a priori information on the prototype category has no significant impact on the performances, as soon as the number for prototypes is high enough. Graphs show mean accuracy (with standard deviation bars) obtained during grid search under the best regularization parameter found. **(Left)** Under L1 regularization, *C* = 10^4^. **(Right)** Under L2 regularization, *C* = 10^2^.

When using the extended metric described in section 3.2.3, we have an extra hyperparameter β weighting the added class-aware term. Based on the previous analysis for *C* and *J*, we decided to constrain the search of *C* ∈ [10^2^, 10^6^] and reduce the number of parameters searched to 3. For *J*, we still search on the same range but limit the number of parameters to 5. Figure 5 shows accuracy results on cross validation for different pairs of β and *J* (with *C* fixed on the best value found by grid search). The contribution of this term for a fixed value of *J* is not clear, at this stage, but it is interesting to notice that when *J* = *N* = 10 there is an improvement until a certain threshold, followed by a plateau (β lower than ≈7.8 under both L1 and L2 regularizations). This can indicate that for large enough β the class-aware term has dominated the positioning of each cluster undermining the influence of the first distance-to-prototypes term. It could also be that for small numbers of prototypes this additional term (that encourages prototypes to cluster samples corresponding to a unique category) has a positive influence on performances, whereas it is no more the case when the number of prototypes is high enough. We consider this result as a good indication that the compromise between a good data representation good classification performances seems possible with our so called data-prototypes.

#### 4.2.2. Interpretability of the Model

**Figure 6:**
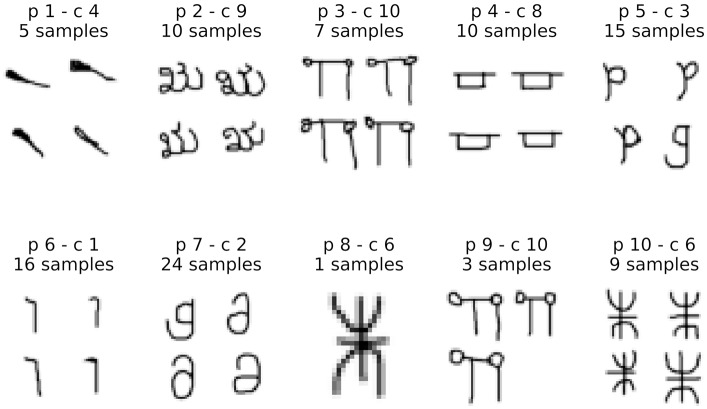
The top-4 closest training samples visually illustrate what is being represented by each prototype. For each prototype p1, p2, .., its related class c2, c5, .. is written, this “main” class being estimated as the most probable class proposed by our algorithm. The number of samples corresponding to the related cluster is given.

**Figure 7:**
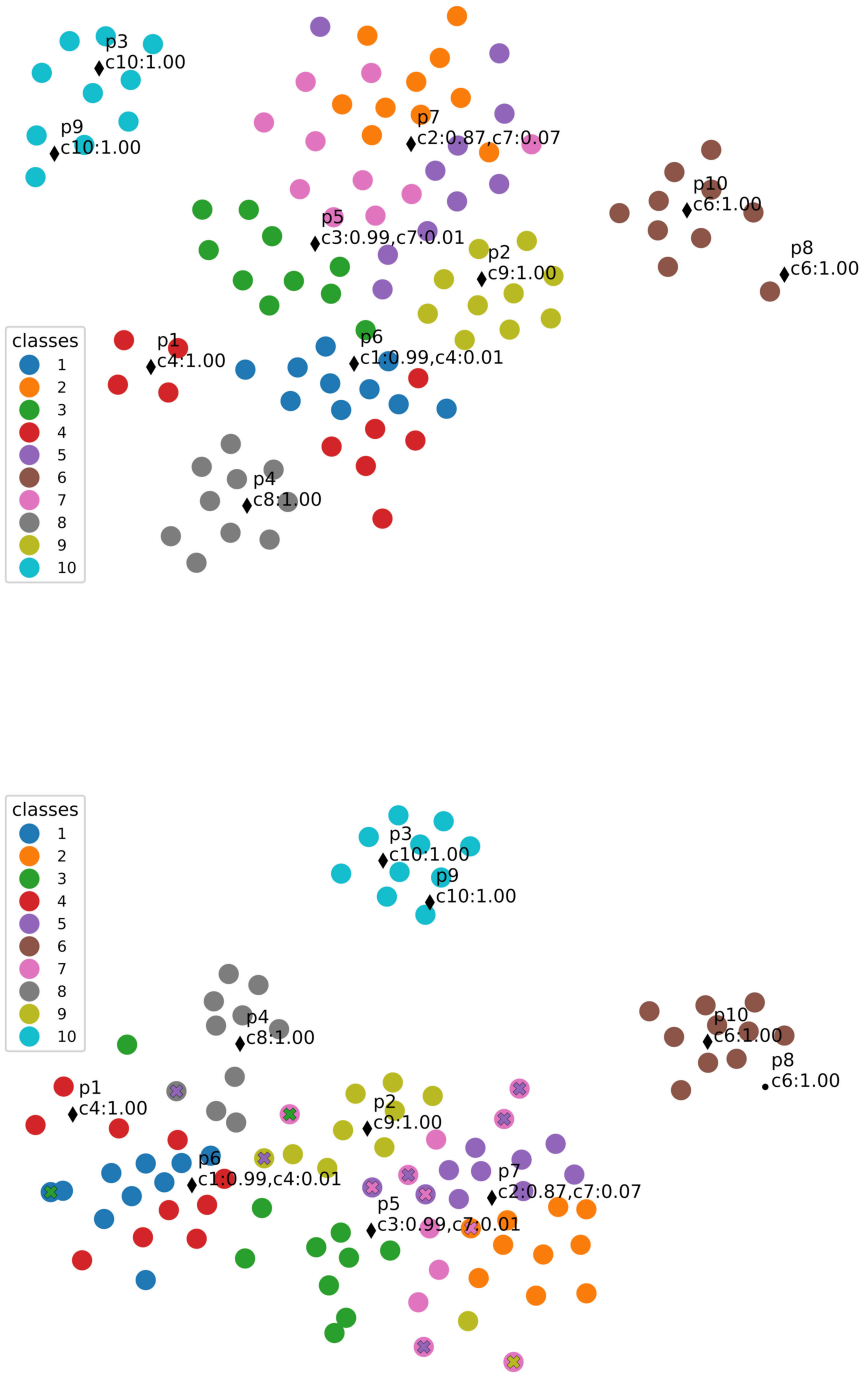
T-SNE visualizations of dataset points (as Inception v3 features) together with learned prototypes, indicated by black markers. Each class is identified by color, and the predicted class for each prototype is annotated. Misclassified points are over-marked by an “x” of the predicted class color. **(Top)** Visualization of the training set; **(Bottom)** Visualization of the test set.

As previously discussed, prototypes in our model lie in the same feature space as the data and correspond to virtual examples. In this section we demonstrate how this fact can support explanations about the model's decisions. For such, we provide a visualization for each prototype by looking at its closest training samples, in Figure 6. To observe prototypes in the feature space, we use T-SNE visualizations of training and test samples in the feature space together with prototypes (Figure 7). Visualizations in this section correspond to our main model combining features and prototypes, under L2 regularization. With this model, since the performance is not sensitive to the number of prototypes, *J* = 10 prototypes was the value chosen by cross validation.

We observe the representative images obtained are coherent with the prototype classes, except for prototype 7, which was classified as class 2 but had a closest sample coming from class 7. This could be due to the fact explained by the fact that both classes correspond to very similar characters (both look like a lowercase “g” under different rotations, see Figure 2), and indeed the T-SNE visualization shows these classes are very mixed in the feature space. Additionally, class 5 is also mixed in and corresponds to a similar character. Indeed, the top-2 most likely classes for p7 predicted with close probability were c2 with 87% and c7 with 7% (see Figure 7), indicating p7 lies in a slightly more ambiguous region of the feature space. In a scenario with an adaptive number of prototypes, this prototype-to-class association information could serve as an index for adding more prototypes in the region.

Representation by the closest training sample illustrates how each prototype can be represented by a corresponding input, having the advantage of being a hyperparameter-free method yielding real images. Of course feature visualization methods based on input optimization could also be applied, but appropriately tuning such models is fundamental for obtaining realistic image representations and out of the scope of this work.

#### 4.2.3. Comparison Over Multiple Episodes

**Figure 8:**
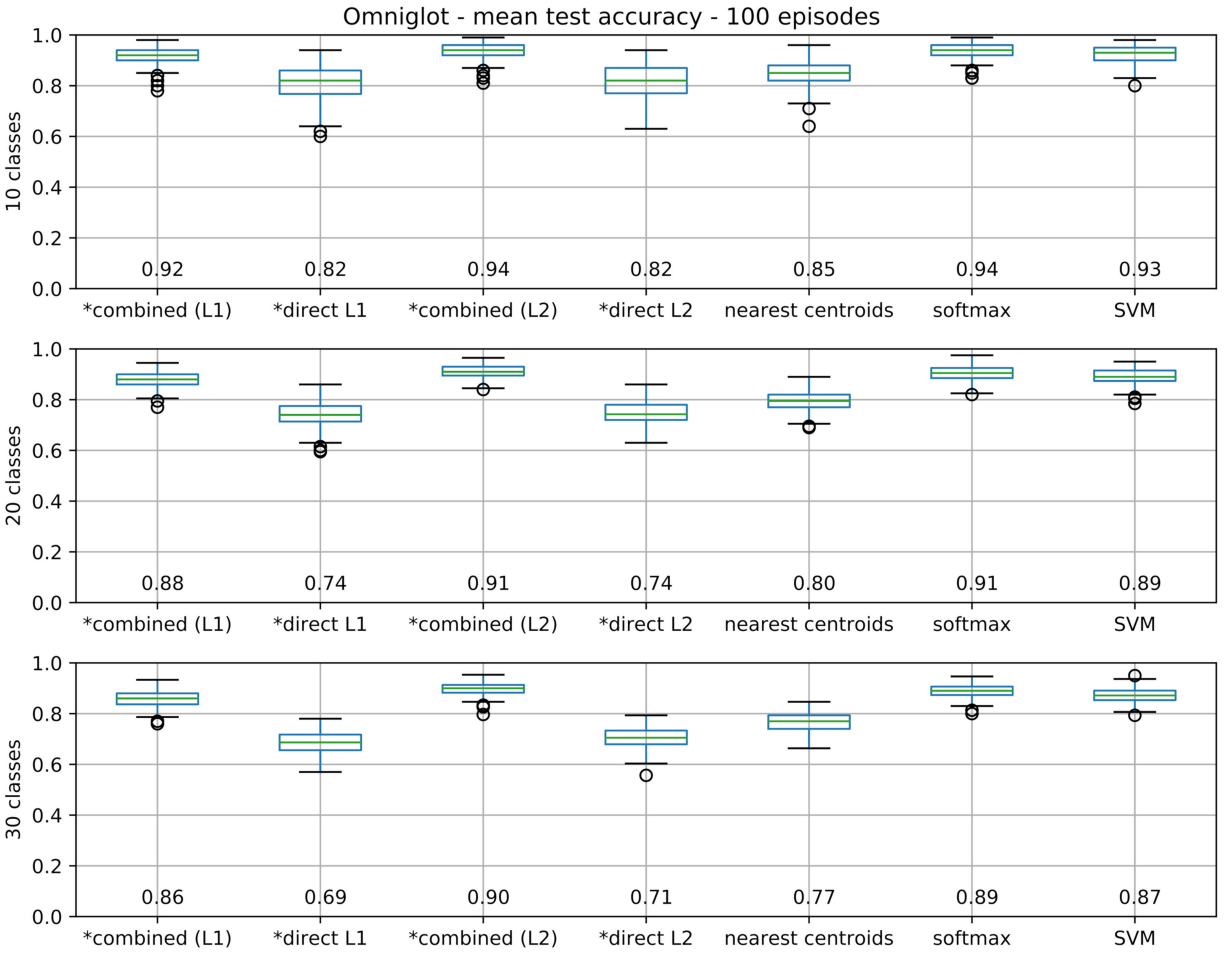
Box plots for test accuracies over 100 episodes. Boxes extend between 1st and 3rd quartiles (*IQR*), with median value noted at the bottom and marked by a green line. Bars mark a range of ±1.5*IQR* from quartiles, with outliers as circles. Our models are marked with a ^*^.

Extending this analysis, we now compare test accuracies over 100 random episodes for different model variants, in order to obtain results of statistical significance. As reference methods we have a softmax regression layer (multinomial regression), an SVM and a nearest class centroids classifier [like the classifier layer used by Snell et al. (2017)]. From our proposal we test four variants: both combined and direct models, using L1 or L2 regularization (as defined in 3.2). Test accuracy results for 100 episodes are displayed in Figure 8.

Statistical significance of the results are verified by a non-parametric Friedman test with *post-hoc* Nemenyi tests (with *p* ≤ 0.05, see Table 1) (Demšar, 2006). No significant differences between both regularizations could be observed for the direct model, while L2 is significantly superior for the combined model. Between direct and combined, combined is significantly better in both cases. Regarding the baseline comparisons, we highlight that the combined model with L2 regularization differs significantly from an SVM for *N* = 20, 30, although no significant difference to softmax could be observed. Additionally, all models differ significantly from nearest-centroids.

**Table 1:** Statistical significance for all pairs of classifiers tested on Omniglot.

**10 classes**						**20 classes**			**30 classes**		
**Models**	**L2**	**Combined L1**	**Combined L2**	**Softmax**	**SVM**	**Softmax**	**SVM**	**L2**	**Softmax**	**SVM**	**L2**
L1	o	++	++	++	++	++	++	o	++	++	o
combined L1	++	–	++	++	o	++	+	++	++	++	++
combined L2	++	++	–	o	o	o	++	++	o	++	++
SVM	++	o	o	++	–	++	–	++	++	–	++

“*++” and “+” mark all pairs which presented a significant difference (for p < 0.01 and p < 0.05, respectively), while “o”s mark those which did not. Non-reported pairs were found to be significantly different with p < 0.01 (except for direct L1 vs. direct L2 at 30 classes, for which p < 0.05)*.

### 4.3. Comparison Over Different Training Sets With Varying Sizes

In order to confirm our results we have considered two different datasets: MNIST and CIFAR10. These datasets have larger training sets, allowing to subsample training sets of size larger than 20*N* (which was the case for Omniglot). Additionally, they have a large standard test set (10 K samples) over which to evaluate the performances. Class distribution in these datasets is approximately balanced, and the distribution is preserved when sampling the training subsets. We explored training subsets of size 100, 200, 300, 500, 750, 1,000 (that is, 10, 20, 30, 40, 50, 75, 100 samples per class on average). The idea is to evaluate performances ranging from few-shot data (here 10 samples per class) to not-so-big data (here a few tenths of samples per class). For hyperparameter selection we proceed exactly as done for Omniglot, by 5 × 2 cross-validation. All models are retrained over the whole training set using the best validation parameters. Statistical significance is also evaluated in the same way (Friedman non-parametric test + Nemenyi post-test). We report the mean test accuracy, averaged over 10 different random training subsets, all evaluated on the standard test partition for each dataset.

**Figure 9:**
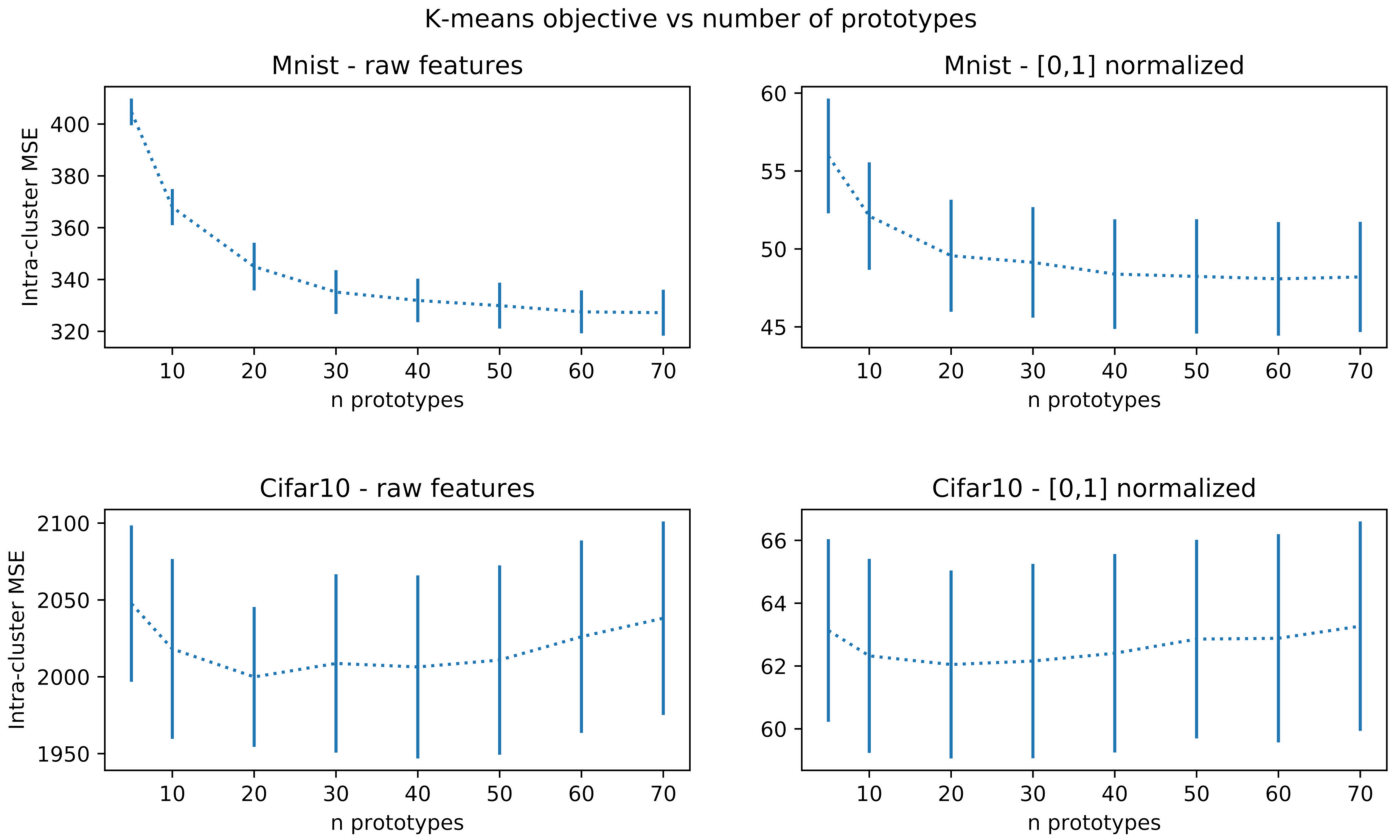
Performances on the k-means algorithm as a function of the number of prototypes, for training subsets with 500 samples from MNIST and CIFAR10 datasets.

We also have numerically studied for these new datasets whether the k-means prototype count automatic adjustment is still numerically valid, as reported in Figure 9, after Figure 4. We thus have now observations for Omniglot (sample 10-shot episode), and MNIST and CIFAR10 (training subset with 500 samples). Such a method indicates 20 clusters for Omniglot and CIFAR10, but a larger number for MNIST (between 30 and 50 depending on a saturation tolerance). The optimal value does not correspond to a minimum but is still detectable as an elbow corresponding to the occurrence of a plateau. This result is not always statistically significant. It is the case for MNIST but not for CIFAR10.

**Figure 10:**
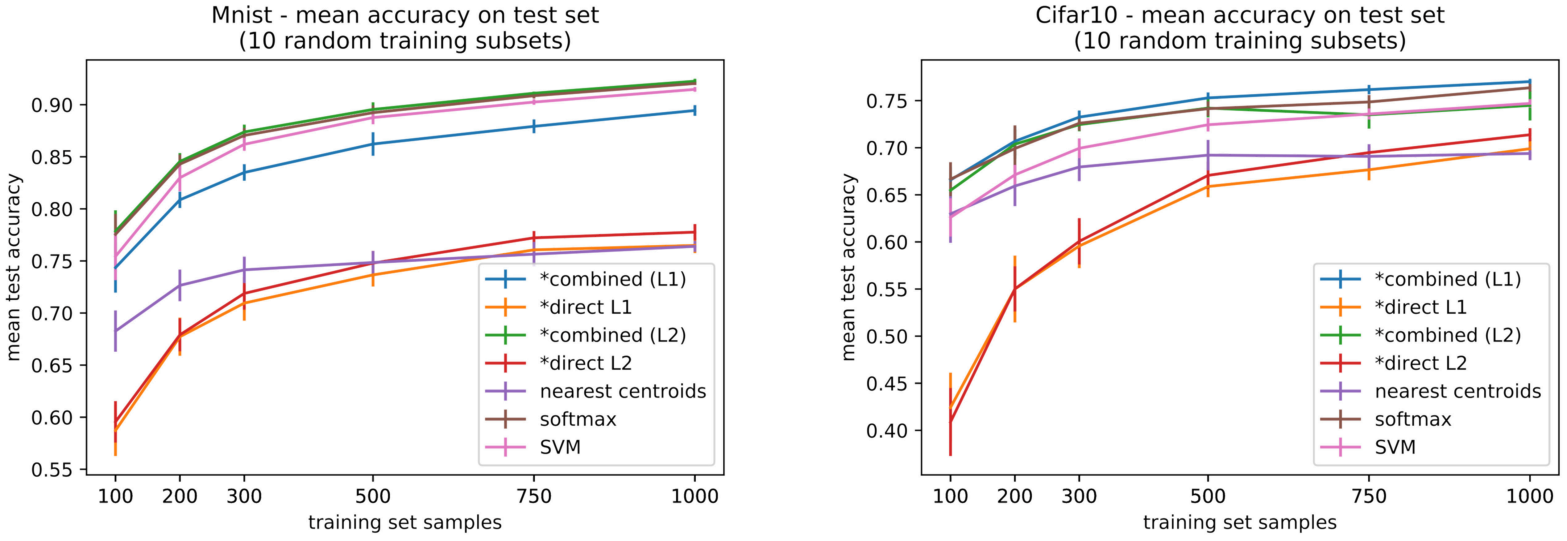
Mean accuracies over standard test sets for MNIST and CIFAR10 datasets, for increasing training set sizes. Results are averaged over 10 different training subsets. Our models are marked with a ^*^.

A first evaluation of the means (and standard deviations, see Figure 10) allows to observe that our combined models perform best or similar to a softmax or an SVM classifier, all performing better than nearest centroids or our direct models, which is congruent with results obtained on Omniglot (but here on a larger test set). Particularly for MNIST, the nearest centroids classifier is considerably lower than the top performing methods.

Considering statistical significance of the comparisons, on MNIST our combined model with L2 regularization is consistently better than nearest centroids, although the same can be said about softmax. In most cases, for both datasets, combined models, softmax, and SVM are significantly better than either of the direct models, but comparison between the two variants (L1 and L2) is inconclusive. For CIFAR10 the significance results are less conclusive, but we do observe some significance in the superiority of the combined model (L1 regularization) over nearest-centroids (between 200 to 1000 training samples). Detailed results for statistical testing of all classifier pairs for all training set sizes can be consulted in the code repository for this paper.

### 4.4. Experiments in the Meta-Learning Paradigm

To allow a fair comparison to usual few-shot learning paradigms, we carried on end-to-end training of the model under meta-learning conditions. We continue to use Omniglot dataset described above, with 4 rotations per character used for data augmentation (0, 90, 270, 360 degrees), and use episodical training, following the methodology of Snell et al. (2017). We use the same CNN architecture (4 layers, 3x3 kernels, resulting in a 64 dimension embedding) and experimental setup, based on their publicly available implementation^9^.

In summary, the task here is to prepare a meta-model which will be able to learn to classify N classes based on k training samples, for different subsets of N classes. For this, the full architecture is meta-learned over a some episodes sampled from a meta-training set containing 1,200 characters, with episodes from a meta-validation set containing 172 characters being used to evaluate and early-stop the meta-training, while the remaining 423 characters are used to generate meta-testing episodes (subsets of N classes not seen in the meta-training phase).

More specifically, the meta-training phase is carried on 100 randomly chosen 5-shot 60-way episodes. Another 100 5-shot 5-way episodes are sampled to form a meta-validation set. Meta-training is stopped after 200 epochs with no improvement on the meta-validation set, keeping the best model so far. At meta-test time, we apply the model to solve 5, 10, 20 and 30-shot learning tasks on 1,000 episodes sampled from previously unseen classes.

Since these experiments are more computationally intensive, using cross-validation to choose hyperparameters becomes prohibitive. We fixed *J* = 2*N*, i.e., 2 prototypes per class and L2 regularization of 10^−4^ (via weight decay). Optimization of the complete model, including CNN weights, is done with an Adam optimizer with initial learning rate of 10^−2^ (and default moment estimation parameters). For each episode the top two layers of model need to be fitted on the new classes using the training (or support) k-shot examples. This optimization was run for 200 iterations, with an initial learning rate of 0.1 (also using Adam). The test (or target) examples serve to evaluate the model and, when in meta-training phase, calculate the loss and gradients that will adjust the bottom CNN weights.

We compare our accuracy results over 1,000 test episodes to two other propositions in the literature: Matching networks (Vinyals et al., 2016) and Prototypical networks (Snell et al., 2017) (see Table 2). These are seminal works in the field of few-shot learning and remain competitive compared to more recent proposals (for instance Garcia and Brina, 2018 or Finn et al., 2017). For prototypical networks, we re-ran the models using the code released by the authors to obtain results for more values of *N* classes. Slightly different results are normal, since we did not do the final retraining step, where the model is retrained in the full meta-training set (including the portion separated for meta-validation).

**Table 2:** Average test accuracy (%) for 5-shot learning on Omniglot over 1,000 episodes.

**Method**	**5 classes**	**10 classes**	**20 classes**	**30 classes**
Matching networks	98.9	–	98.5	–
Matching networks – fine tuning	98.7	–	98.7	–
Prototypical networks	99.7	–	98.9	–
Prototypical networks (re-run)	99.54 ± 0.07	99.17 ± 0.06	98.57 ± 0.06	98.01 ± 0.06
Direct - 2N prototypes	98.4 ± 0.2	97.2 ± 0.2	94.8 ± 0.3	92.6 ± 0.4
Combined - 2N prototypes	97.1 ± 0.2	96.3 ± 0.2	96.2 ± 0.1	95.9 ± 0.1

*We compare to results reported by two other works: Matching networks (Vinyals et al., 2016) and Prototypical networks (Snell et al., 2017)*.

Overall, we can observe that learning the CNN weights through this meta-training approach yields better performances in comparison to using fixed features, as expected. Considering that no hyperparameter tuning was done, we still obtain good results, a little lower than the state-of-the-art, even though they degrade faster as the number of classes goes up. Some discussion on this result is presented in the next section. Between combined and direct model, we also observe that the former performs better at a higher number of classes *N* = 20, 30, while the second is better for *N* = 5, 10.

## 5. Discussion

### 5.1. Performances and Use of the Algorithm

We have proposed a simple three-step model, applied to classification tasks on visual data under very small (few-shot on Omniglot) to not-so-big datasets (subsets from MNIST or CIFAR10). Let us now discuss the main questions presented in the course of this work in the light of our experimental results.

- *Is the setup able to properly learn such a task in an interpretable way?* Our models were capable of performing the classification tasks, both under fixed features and under the meta-learning paradigm, while profiting from the explainability support that can be provided by our rather simple architecture. Our “data prototypes” that can be used to provide a visual explanation of the model's internal representations, by indicating ambiguous classification regions for example. Some mathematical discussions on how the prototypes induce a partition of the feature space are presented in Appendices A5, A6. This explanatory power is however limited when using the combined model, since raw features are also considered in prediction. Prioritizing interpretability by using only the direct model yields reduced performances under fixed features, indicating a trade-off between interpretability and classification accuracy in this case.

Altogether, this is no more than a variant of the notions of prototypes used in the field, but with the objective to both represent the data in an unsupervised way and the supervised information. The name “data prototype” is chosen particularly because they may be considered as virtual samples that summarize what has been learned during the learning phase, acting as a local descriptor in the feature space. Being virtual samples, they could be fed to feature visualization techniques to provide a graphic explanation (here exemplified by taking the closest training samples as a visualization proxy).

Another non negligible aspect is the fact that our proposed architecture, schematized in Figure 1 is “easy to explain” to non-specialists : (i) features are extracted (by the deeper layers), (ii) the data is organized in regions (parameterized by prototypes), and (iii) the classification is performed combining all information. Our will to provide an interpretable and understandable process is not only a wish but corresponds to real interaction with large public targets, including providing didactic resources on these subjects, as reported in Drumond et al. (2017a) or available in Chraibi Kaadoud and Viéville (2016).

- *To which extents does the prototype dimensional reduction impairs the global performances?* Under fixed features, indeed we have a strong dimensionality reduction which impairs the performance of our direct model, which justifies the use of a combination of features and prototype information in our main model. This way it is possible to both benefit from the prototype layer and obtain optimized performances as good as or better than a standard method.

In meta-learning paradigm, we also learn the features by learning the weights of a CNN that feeds our model. In this case, since the features are learned together with our prototype model, the class representations should become more adapted to a prototype-based discrimination, resulting in higher accuracies than under fixed features even for the direct model. Moreover, the dimensionality reduction was not as strong because we use a 64 dimension embedding and 2*N* prototypes.

Combining these two options allows the user to both obtain state of art performances and interpretable results. With respect to other comparable existing methods, we have obtained similar performances, except when considering meta-learning with a large number of classes. This is explained by the fact that we have not optimized hyper-parameters in this case, which had already been done in the fixed-features paradigm.

We can also hypothesize that our algorithm, since based on a reduced set of prototypes that acts as support-vectors of the classification (see Viéville and Crahay, 2004, for a development of the link between support-vectors and prototypes), may have good generalization properties, e.g., may limit overfitting which is to be expected in a not-so-big data setup. This is indeed the case for the direct model. For the combined model, as detailed in Appendix A7, we may interpret the softmax layer as piece-wise linear classifier considering *O*(*L*^2^) prototypes. As a perspective of this work, going in depth in this interpretation may be an interesting issue.

- *Is the hyperparameter adjustment automatic at the end-user level?* The main hyperparameters we introduce are the number of prototypes and a typical regularization strength, this last one being not too critic (specially for the combined model). Even if the number of prototypes is a non-negligible choice in terms of data representation, with the combined model we eliminate sensitivity to this hyper parameter in terms of performance. However, in terms of interpretability, this leads to choosing a small number of prototypes that might no represent so well classes in the feature space. Conversely, with the direct model, we are led to choose an always large number of prototypes at the risk of overfitting, because at the limit (one prototype by learning sample) the method reduces to a nearest-neighbor classifier, known to be of poor generalization performances. Here, we fix this issue by proposing to choose the number of prototypes in the clustering phase based directly on its unsupervised loss, optimizing it for data representation. We have experimentally verified the feasibility of this method for the different data sets. Comparing to other methods, this seems to be the main new qualitative result to point out.

### 5.2. Perspectives and Future Work

Beyond these outcomes, general or application dependent extensions of the method can be pointed out.

+ *Consider incremental learning*, where learning can continue when given new samples, by updating the prototypes and the related category prediction. This can be achieved for instance through incremental k-means, which is a standard process (see, e.g., Aaron et al., 2014). Applying cross-entropy minimization in an incremental manner is also straightforward, iteratively adding loss terms for newly arrived samples.

+ *Consider prototype editing*, where (i) redundant prototypes within the same connected component can be deleted, while (ii) learning samples detected as exceptions and generating a classification error can be taken into account via a new prototype. Additionally, and adaptive mechanism for determining the number of prototypes could better explain the data representation, improving interpretability of the feature space, while eliminating one sensitive hyperparameter of the model. In domain-specific applications where classes might be composed of many different subclasses, a sensible number of prototypes could better identify these subclasses and provide means of representing the different subgroups.

+ *Interacting with the deep-learning lower layers*, since the knowledge of the prototypes may help disentangling the lower-layer feature extraction. A class disentanglement is a likely cause behind the improved performances in the meta-learning setting (when compared to using fixed features), since all layers are trained end-to-end. In a transfer learning setting, we could proceed to the fine-tuning of the upper layers of the pre-trained CNN to try to achieve a similar result. Moreover, disentanglement could be enforced implicitly via low dimensional manifold regularization (Zhu et al., 2018), or using generative latent factor models (Hoogeboom, 2017). Another form of interaction could be the introduction of shortcuts from lower layers to the feature layer in order to increase the prototype components, analogous to other architectures incorporating some form of forward shortcut (He et al., 2016; Shelhamer et al., 2016; Huang et al., 2017).

+ *Integrate knowledge from unlabeled examples* is also a possibility. These examples can represented in the same feature space as other labeled samples and prototypes, using same embedding CNN to extract this representation. These examples then can be taken into account when computing the prototype coordinates, analogously to what has been demonstrated by Ren et al. (2017). Particularly when trying to solve ambiguous regions where the system makes mistakes, a related track is to develop some active learning heuristics and infer which new examples could be of most help if labeled.

## 6. Conclusion

We analyzed how considering data prototypes at a different level with respect to the state of the art allows to both reasonably work with small data sets, and with an interpretable view of the obtained results. The proposed architecture is minimal and combine both the performances of a standard method and the capability to observe the feature space representation.

At the methodological level, we also pay attention at exploring in details the performances and limitations of the proposed set-up, in an analogous way as biological experiments are conducted, considering in details all hyperparameters involved in the process. This is somehow different from simply “winning the game” with respect to some deep learning benchmarks.

## Author Contributions

TD is the main author at the origin of the data prototype idea and has designed, developed and performed the numerical experiments and their analysis. FA, TV, and TD wrote sections of the manuscript. All authors contributed to manuscript revision, read and approved the submitted version.

### Conflict of Interest Statement

The authors declare that the research was conducted in the absence of any commercial or financial relationships that could be construed as a potential conflict of interest.

## Footnotes

^1^Here compact means that adding a network layer allows a better input-output mapping approximation or a mapping considering a higher functional sub-space for a number of units which would have been exponentially higher with decreasing the number of layers.

^2^Further aspects of feed-back functionality, such as on-line computations with feed-backs, will not be considered here.

^3^The notions of “automated machine learning,” “meta-learning,” including “meta-optimization” and “hyperparameter” and the related “hyperparameter optimization,” have precise meaning in machine learning, and we assume this is known to the reader.

^4^Not to be confused with meta-learning in the sense of hyperparameter tuning and automatic machine learning.

^5^**Notations:** Vectors and matrices are written in bold, the vector dot-product writes **x**^*T*^**x**′, i.e., as a matrix product with the row vector transpose of the left column vector. The Heaviside function writes *H*(*u*). Partial derivatives are written in compact form, e.g., ∂_*x*_*n*__**f**(**x**) means $\frac{\partial \text{f}\left(\text{x}\right)}{\partial x_{n}}$. The notation δ_P_ stands for 1 if the property P is true and 0 otherwise (e.g., δ_2>1_ = 1). The notation {*a, b*} stands for an integer interval and [*a, b*] for a real interval. When considering a vector **x** augmented by new dimension **c** we simply write (**x**, **c**) for the corresponding element in the Cartesian product of the corresponding spaces.

^6^Obviously, if the classification task is performed on $\tilde{\text{x}}$, i.e., if we consider $c_{l}(\text{x})=\nu_{l}\left({\text{w}}_{l}^{\prime T}\tilde{x}\right)$, this is equivalent to the previous form $c_{l}(\text{x})=\nu_{l}\left({\text{w}}_{l}^{T}\text{q}\left(\text{x}\right)\right)$, with ${\text{w}}_{l}^{\prime T}{\text{p}}_{j}={\text{w}}_{l}^{T}$, thus simply correspond to an over-parameterization of the classification layer using *D* × *N* weights, but with only *J* × *N* degrees of freedom.

^7^http://yann.lecun.com/exdb/mnist

^8^https://www.cs.toronto.edu/ kriz/cifar.html

^9^Code for prototypical networks available at https://github.com/jakesnell/prototypical-networks, under MIT license. Our fork is available at https://github.com/thalitadru/prototypical-networks
