# Supplementary material for: Bio-inspired Analysis of Deep Learning on Not-So-Big Data Using Data-Prototypes
Source: Front Comput Neurosci. 2019 Jan 9;12:100. doi: 10.3389/fncom.2018.00100 (PMC6333865; doi:10.3389/fncom.2018.00100)
Supplement: Supplementary file 1 [file Data_Sheet_1.pdf]

# Appendix

## APPENDIX A: MODEL DERIVATION AND FORMAL INTERPRETATION

### A1: Sample to prototype association

We approximate the probability $q_j(\mathbf{x})$ for the feature vector $\mathbf{x}$ of a sample, to be associated to the $j$-th prototype writing:

$$\begin{array}{rcll} q_j(\mathbf{x}) & \stackrel{\text{def}}{=} & {}_\tau\nu_j\left(-\|\mathbf{x}-\mathbf{p}_j\|^2/2\right) & \\ & = & {}_\tau\nu_j\left(\mathbf{p}_j^T\,\mathbf{x} - \|\mathbf{p}_j\|^2/2 - \|\mathbf{x}\|^2/2\right) & \text{by expansion} \\ & = & {}_\tau\nu_j\left(\mathbf{p}_j^T\,\mathbf{x} - \|\mathbf{p}_j\|^2/2\right) & \text{by offset invariance} \end{array}$$

using the soft-max function:

$${}_\tau\nu_j\left(\mathbf{x}\right) \stackrel{\text{def}}{=} \frac{\exp(f_j(\mathbf{x})/\tau)}{\sum_{j'}\exp(f_{j'}(\mathbf{x})/\tau)}$$

writing $f_j(\mathbf{x}) \stackrel{\text{def}}{=} \mathbf{p}_j^T\,\mathbf{x} - \|\mathbf{p}_j\|^2/2$, thus $\partial_{\mathbf{p}_j} f_j(\mathbf{x})^T = \mathbf{x} - \mathbf{p}_j$. In the core of the paper we omit the temperature $\tau$ without loss of generality since integrated in the $f_j(\cdot)$ parameters. See Appendix A2 for a review of the basic equations used in this paper.

More generally we could consider $f_j(\mathbf{x}) \stackrel{\text{def}}{=} -\|\mathbf{x}-\mathbf{p}_j\|^d/d$, yielding $\partial_{\mathbf{p}_j} f_j(\mathbf{x}) * T = \|\mathbf{x}-\mathbf{p}_j\|^{d-2}\,(\mathbf{x}-\mathbf{p}_j)$, in order to deal with more than the $\mathcal{L}^2$ norm (e.g., for sparse estimation).

### A2: Remainder on softmax function

The softmax function generalizes the logistic function and enjoys the facts that:

$$0 \leq {}_\tau\nu_j\left(\mathbf{x}\right) \leq 1,\; \textstyle\sum_j {}_\tau\nu_j\left(\mathbf{x}\right) = 1,\; {}_\tau\nu_j\left(\mathbf{x}\right)_{f_j \rightarrow f_j + o} = {}_\tau\nu_j\left(\mathbf{x}\right),\; {}_{\tau/\tau'}\nu_j\left(\mathbf{x}\right) = {}_\tau\nu_j\left(\mathbf{x}\right)^{\tau'} \Big/ \textstyle\sum_{j'} \nu_{j'}\left(\mathbf{x}\right)^{\tau'},$$

in words, yields probability distributions with with offset invariance and power effect of the temperature $\tau$, while:

$${}_\tau\nu_j\left(\mathbf{x}\right) = \max_j(f_j(\mathbf{x}))_{\tau \rightarrow o} = \tfrac{1}{J} + \tfrac{1}{\tau\,J}\left(f_j(\mathbf{x}) - \tfrac{1}{J}\textstyle\sum_{j'} f_{j'}(\mathbf{x})\right) + O\left(\tfrac{1}{\tau^2}\right),$$

computes the maximal values at low temperature, while approximating the given affine operator at high temperature. Moreover, for some parameter $\theta_{j'}$ of the $f_{j'}(\mathbf{x})$ we obtain:

$$\tau\,\partial_{\theta_{j'}\,\tau}\nu_{j'}(\mathbf{x}) = {}_\tau\nu_j(\mathbf{x})\left(\delta_{j=j'} - {}_\tau\nu_{j'}(\mathbf{x})\right)\partial_{\theta_{j'}} f_{j'}(\mathbf{x}),$$

while for some parameter $\theta$ of the $\mathbf{x}$ we obtain:

$$\tau\,\partial_{\theta\,\tau}\nu_j(\mathbf{x}) = {}_\tau\nu_j(\mathbf{x})\left(\partial_\mathbf{x} f_j(\mathbf{x}) - \textstyle\sum_{j'} {}_\tau\nu_{j'}(\mathbf{x})\,\partial_\mathbf{x} f_{j'}(\mathbf{x})\right)\partial_\theta \mathbf{x}.$$

Considering an approximate cross-entropy criterion to adjust the values of a standard soft-max criterion yields the following minimization:

$$\begin{array}{rcll} \mathcal{C} & = & -\int_\mathcal{X} p(\mathbf{x})\,\log\left(\nu(\mathbf{x})\right) & \\ & \simeq & -\frac{1}{N}\sum_i \log\left(\nu(\mathbf{x}_i)\right) & \text{approximating } p() \text{ on the data samples} \\ & = & -\frac{1}{N}\sum_i \log\left(\nu_{l_i}(\mathbf{x}_i)\right) & \text{since each sample is associated to one class} \\ & = & -\frac{1}{\tau\,N}\sum_i f_{l_i}(\mathbf{x}_i) + \log\left(\sum_{j'}\exp(f_{j'}(\mathbf{x})/\tau)\right) & \text{by substitution and factorization} \end{array}$$

yielding the gradient, for a parameter $\theta_{j'}$ of the function $f_{j'}(\mathbf{x})$:

$$-\tau\,\partial_{\theta_{j'}}\mathcal{C} = \left(\tfrac{1}{N}\textstyle\sum_i \delta_{j'=l_i} - {}_\tau\nu_{j'}(\mathbf{x})\right)\partial_{\theta_{j'}} f_{j'}(\mathbf{x}).$$

## A3: Deriving the model variational equations

Considering an approximate cross-entropy criterion to adjust the parameters given samples $\mathbf{x}_i$ and their label $l_i$ yields the following minimization:

$$\begin{array}{rcl} \mathcal{C} & = & -\int_{\mathcal{X}} p(\mathbf{x}) \, \log\left(c(\mathbf{x})\right) \\ & \simeq & -\frac{1}{N} \sum_i \log\left(c_{l_i}(\mathbf{x}_i)\right) \quad \text{approximating } p() \text{ on the data samples} \end{array}$$

yielding:

$$\begin{array}{rcl} -N \, \tau \, \partial_{\mathbf{w}_l} \mathcal{C}^T & = & \sum_i \underbrace{\left[\delta_{l=l_i} - c_l(\mathbf{x}_i)\right]}_{\zeta_i} \, \mathbf{q}(\mathbf{x}_i) \\ -N \, \tau \, \partial_{\mathbf{p}_{j'}} \mathcal{C}^T & = & \sum_i \underbrace{\left[\sum_j \left(w_{l_i j} - \sum_l c_l(\mathbf{x}_i) \, w_{lj}\right) q_j(\mathbf{x}_i) \, \left(\delta_{j=j'} - q_{j'}(\mathbf{x}_i)\right)\right]}_{\xi_{ij'}} \quad (\mathbf{x}_i - \mathbf{p}_{j'}) \end{array}$$

so that we can write, for some sufficiently small $\epsilon$:

$$\Delta \mathbf{w}_l = \epsilon \sum_i \zeta_i \, \mathbf{q}(\mathbf{x}_i) \text{ and } \mathbf{p}_j = \sum_i \xi_{ij} \, \mathbf{x}_i / \sum_i \xi_{ij}$$

at each step, and leading to:

- expectation (estimated the prototypes as weighted mean over the samples),
- minimization (decreasing the criterion by adjusting the soft-max weights) mechanism.

With such an approach there is a weak clustering of the samples with respect to a prototype, e.g., samples with different labels can be related to the same prototype.

## A4 : Analysis of the k-means extended metric

Let us consider the for a given sample $\mathbf{x}_i$ the augmented feature space of dimension $D + J$: $(\mathbf{x}_i, \mathbf{c}_i)$ where $\mathbf{c}_i \in [0, 1]^J$ are the a-priori probabilities for the sample of index $i$ to belong to each category. For a learning sample $(\mathbf{x}_i, l_i)$ of known category $l_i$ we obviously have $c_{ik} = \delta_{l_i = k}$. Such augmented dimensions act as a level-set over the feature space.

Let us consider a standard k-means algorithm on such extended feature space, for some $\beta > 0$:

$$(\mathbf{p}_j, \mathbf{c}_j) = \sum_i \xi_{ij} \, (\mathbf{x}_i, \mathbf{c}_i) / \sum_i \xi_{ij}, \text{ with } j_{\mathbf{x}} = \arg\min \|\mathbf{x}_i - \mathbf{p}_j\|_2 + \beta \, \|\mathbf{c}_i - \mathbf{c}_j\|^2$$

with $\xi_{ij} = \delta_{j=j_{\mathbf{x}}} \in \{0, 1\}$ for a hard k-means algorithm, while more general soft k-means mechanism with $\xi_{ij} \geq 0$ (as in the previous subsection) could be introduced, the prototype being the centroid of the samples belonging to this cluster.

If $\beta = 0$ the augmented dimensions are not taken into account, and we are left with the original k-means mechanism.

If $2 \, \beta > M \stackrel{\text{def}}{=} \max_{ii'} |\mathbf{x}_i - \mathbf{x}_{i'}|^2$ then it is easy to verify that two samples with different categories can not be in the same cluster, providing that the number of prototype is not lower than the number of category.

To verify this fact, let us consider two samples $(\mathbf{x}_i, l_i)$ and $(\mathbf{x}_{i'}, l_{i'})$ with $\mathbf{x}_i \neq \mathbf{x}_{i'}$ and $l_i \neq l_{i'}$ so that

$$\|\mathbf{c}_i - \mathbf{c}_{i'}\|^2 = \sum_k (\delta_{l_i = k} - \delta_{l_{i'} = k})^2 = 2$$

since for $k = l_i$ and $k = l_{i'}$ the values in the summation differs by a value of 1. As a consequence, regarding their extended distance

$$\|\mathbf{x}_i - \mathbf{x}_{i'}\|_2 + \beta \, \|\mathbf{c}_i - \mathbf{c}_{i'}\|^2 = \|\mathbf{x}_i - \mathbf{x}_{i'}\|_2 + 2\beta > M$$

it is higher than any sample pair within the same category. Furthermore, if a prototype corresponds to samples of the same category its within-cluster maximal square distance to each sample is lower than $M$, as being the centroid of the samples belonging to this cluster. As consequence, as soon as two prototypes are used in the algorithm, if these two samples are in

the same cluster, the within-cluster distance is going to be higher than any solution with clusters only grouping samples of the same category.

Therefore, considering a k-means algorithm with a proper initialization mechanism that minimizes the within-cluster distance, we have a mechanism that weight the importance of taking the a-priori information about category into account.

## A5 : Probabilistic interpretation of representing samples by prototypes

Representing the sample $\mathbf{x}$ by prototypes means approximating the $\mathbf{x}$ distribution by a distribution only function of the prototypes, e.g.:

$$\mathbf{x} \simeq \mathbb{E}_{\hat{q}}(\mathbf{x}) = \sum_j q_j(\mathbf{x})\, \mathbf{p}_j, \text{ with } \hat{q}(\mathbf{x}) \stackrel{\text{def}}{=} \sum_j q_j(\mathbf{x})\, \delta(\mathbf{x} - \mathbf{p}_j)$$

where $\hat{q}(\mathbf{x})$ approximates the true sample probability distribution $p(\mathbf{x})$ as a discrete distribution, given the prototypes.

Another view is to consider a partition $\{\cdots, P_j, \cdots\}$ of the space induced by the prototypes (i.e., with $\mathbf{p}_j \in P_j$, see Appendix C), yielding:

$$q(\mathbf{x}_i) = \sum_j p(\mathbf{x}_i \in P_j)\, p(\mathbf{p}_j|\mathbf{x}_i) = \sum_j \kappa_{ij}\, q_j(\mathbf{x}_i)$$

for some $\kappa_{ij} = p(\mathbf{x}_i \in P_j)$. Here the partition has not to be made explicit, only the $\kappa_{ij}$ have to be estimated.

Very easily, we obtain $\sum_j \kappa_{ij} = 1$ (since $P_j$ forms a partition), while the $\kappa_j \stackrel{\text{def}}{=} \sum_i \kappa_{ij}$ is the expectation of the number of sample in the partition, i.e. associated to the prototype. If $\kappa_j = 0$ the prototype is inactive, i.e., not associated to any sample. If $\kappa_{ij} \in \{0, 1\}$, i.e, if we know whether $\mathbf{x}_i \in P_j$ or not, i.e., is related to this prototype or not. In such a situation, the constraint $\sum_j \kappa_{ij} = 1$ states that each sample is associated to a unique prototype, while $\kappa_j \stackrel{\text{def}}{=} \sum_i \kappa_{ij}$ is the number of samples associated to a given prototype. This restrained modeling is not what is proposed in this paper.

Moreover, the fact we consider for $c_l(\mathbf{x})$:

$$p(l|\mathbf{x}) = {}_{\tau}\nu_l \left(\mathbf{w}_l^T\, p(\mathbf{p}_j \in Q_j|\mathbf{x})\right) \neq \sum_j p(l|\mathbf{p}_j \in Q_j)\, p(\mathbf{p}_j \in Q_j|\mathbf{x})$$

simply means that we do *not* consider that $Q_j$, namely the set of samples associated to the $j$-th prototype, corresponds to a partition of the sample space $\mathcal{X}$, but that the corresponding regions may overlap, while some regions may not correspond to any samples associated to any prototypes.

## A6: Duality between partition, prototypes and metric

Given a set of prototypes $\mathbf{P} = \{\cdots \mathbf{p}_j \cdots\}$ in a topological space $\mathcal{X}$ we can consider a partition of the space $P = \{\cdots P_j \cdots\}$ around the prototypes, i.e. such that

$$\mathbf{p}_j \in P_j \neq \emptyset, \quad \cup_j P_i = \mathcal{X}, \quad \forall i, j, \mathring{P}_j \cap \mathring{P}_i = \emptyset.$$

The last condition means that we do not require the intersection to be empty but only its interior (i.e., maximal open set). This notion of partition is thus related to a topology. Roughly speaking, this means that we do not take into account what happens at the frontier between two subsets of the partition. For any point, but those at the frontier between two subsets, we can define its partition subset index $j = p(\mathbf{x}) = \{j, \mathbf{x} \in P_j\}$.

Given a set of prototypes, any metric induces such a partition, writing:

$$P_j = \{\mathbf{x} \in \mathcal{X}, d(\mathbf{x}, \mathbf{p}_j) <= min_{j'} d(\mathbf{x}, \mathbf{p}_{j'})\}$$

On the reverse, given a partition and a topology, the ultrametric:

$$d(\mathbf{x}, \mathbf{y}) = \delta_{\mathbf{x} \neq \mathbf{y}}\, (1 + \delta_{p(\mathbf{x}) \neq p(\mathbf{y})}) \in \{0, 1, 2\}$$

is a trivial metric compatible with the partition (i.e. fitting in the previous definition).

More interesting is the fact that given any partition with a metric, and choosing a precision $\epsilon$ there exists a countable set of prototypes such that the given partition is a subset of the partition induced by these prototypes, up to the $\epsilon$ precision. If $\mathcal{X}$ is bounded, e.g. compact, the prototype set if finite. Let us consider this case. Any compact set has always a finite cover. As being a metric space, it always has a finite cover by balls of a given radius, say $B(\mathbf{p}_i, \epsilon/2)$. From this construction, we only keep the prototypes corresponding to the center of balls that intersect the initial partition subsets frontiers. It is then easy to prove that this induce, up to $\epsilon$, a partition:

$$\mathbf{p}_j \in P_j \neq \emptyset, \quad \cup_j P_i = \mathcal{X}, \quad \forall i, j, P_j \cap P_i \subset B(\mathbf{p}_i, \epsilon),$$

where $P_j$ is defined from the metric as given above. Such a construction is related to vector support machines, the given prototypes being somehow the classification support set.

## A7: Relation between softmax and prototypes

In our model specification we relate a prototype representation to a softmax function writing

$$q_j(\mathbf{x}) = \nu_j \left(-\|\mathbf{x} - \mathbf{p}_j\|^2/2\right).$$

Let us develop here the inverse relation and see to which extent we can link a softmax function to prototypes.

Consider an affine softmax function:

$${}_\tau\nu_l\,(\mathbf{x}) \stackrel{\text{def}}{=} \frac{\exp(f_l(\mathbf{x})/\tau)}{\sum_{j'} \exp(f_{j'}(\mathbf{x})/\tau)}$$

with $f_l(\mathbf{x}) \stackrel{\text{def}}{=} \mathbf{w}_l^T\,\mathbf{x} + w_l^0$ and the decision rule

$$l_{\mathbf{x}} = \text{arg max}_{l'}\,{}_\tau\nu_{l'}\,(\mathbf{x})$$

This yields a piece-wise linear segmentation of the feature space[1]. Writing $\mathbf{w}_{ll'} = \mathbf{w}_l - \mathbf{w}_{l'}$ we obtain:

$$l_{\mathbf{x}} = \{l, \forall l' \neq l, \mathbf{w}_{ll'}^T\,\mathbf{x} + w_{ll'}^0 \geq 0\}$$

for the $N\,(N-1)/2$ category pairs.

For each inequality we may consider any pair of points $\mathbf{p}_{ll'}, \mathbf{p}_{l'l}$ for which the separation hyperplane defined by $\mathbf{w}_{ll'}^T\,\mathbf{x} + w_{ll'}^0 = 0$ is the median. We can this write[2]:

[1] Since:

$$\begin{aligned}
& {}_\tau\nu_l\,(\mathbf{x}) \gtrless {}_\tau\nu_{l'}\,(\mathbf{x}) \\
\Leftrightarrow\; & \frac{\exp(f_l(\mathbf{x})/\tau)}{\sum_{l''} \exp(f_{l''}(\mathbf{x})/\tau)} \gtrless \frac{\exp(f_{l'}(\mathbf{x})/\tau)}{\sum_{l''} \exp(f_{l''}(\mathbf{x})/\tau)} \\
\Leftrightarrow\; & \exp(f_l(\mathbf{x})/\tau) \gtrless \exp(f_{l'}(\mathbf{x})/\tau) \\
\Leftrightarrow\; & f_l(\mathbf{x}) \gtrless f_{l'}(\mathbf{x})
\end{aligned}$$

the denominators being identical and the exponential being a strictly increasing function, we make explicit this piece-wise linear segmentation.

[2] We easily derive:

$$\begin{aligned}
& d(\mathbf{x}, \mathbf{p}_{ll'}) \lessgtr d(\mathbf{x}, \mathbf{p}_{l'l}) \\
\Leftrightarrow\; & d(\mathbf{x}, \mathbf{p}_{ll'})^2 \lessgtr d(\mathbf{x}, \mathbf{p}_{l'l})^2 \\
\Leftrightarrow\; & \|\mathbf{x}\|^2 - 2\mathbf{p}_{ll'}^T\,\mathbf{x} + \|\mathbf{p}_{ll'}\|^2 \lessgtr \|\mathbf{x}\|^2 - 2\mathbf{p}_{l'l}^T\,\mathbf{x} + \|\mathbf{p}_{l'l}\|^2 \\
\Leftrightarrow\; & -2(\mathbf{p}_{ll'} - \mathbf{p}_{l'l})^T\,\mathbf{x} + \|\mathbf{p}_{ll'}\|^2 - \|\mathbf{p}_{l'l}\|^2 \lessgtr 0 \\
\Leftrightarrow\; & -4\,\lambda\,\mathbf{w}_{ll'}^T\,\mathbf{x} + \|\mathbf{p}_{ll'}\|^2 - \|\mathbf{p}_{l'l}\|^2 \lessgtr 0 \\
\Leftrightarrow\; & -4\,\lambda\,\mathbf{w}_{ll'}^T\,\mathbf{x} - 4\,\alpha\,\lambda\|\mathbf{w}_{ll'}\|^2 \lessgtr 0 \\
\Leftrightarrow\; & -4\,\lambda\,\left(\mathbf{w}_{ll'}^T\,\mathbf{x} + w_{ll'}^0\right) \lessgtr 0 \\
\Leftrightarrow\; & \mathbf{w}_{ll'}^T\,\mathbf{x} + w_{ll'}^0 \gtrless 0
\end{aligned}$$

.

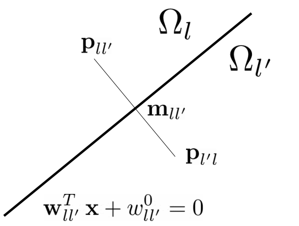


$$\begin{array}{rcl}
\mathbf{m}_{ll'} & \stackrel{\mathrm{def}}{=} & \mathbf{w}_{ll'}^{\perp} - \alpha\,\mathbf{w}_{ll'} \\
\mathbf{p}_{ll'} & \stackrel{\mathrm{def}}{=} & \mathbf{m}_{ll'} + \lambda\,\mathbf{w}_{ll'} \\
\mathbf{p}_{l'l} & \stackrel{\mathrm{def}}{=} & \mathbf{m}_{ll'} - \lambda\,\mathbf{w}_{ll'} \\
\alpha & \stackrel{\mathrm{def}}{=} & w_{ll'}^0 / \|\mathbf{w}_{ll'}\|^2
\end{array}$$

for any $\lambda > 0$ and any vector $\mathbf{w}_{ll'}^{\perp}, \mathbf{w}_{ll'} \perp \mathbf{w}_{ll'}^{\perp} = 0$. These pairs of points $\mathbf{p}_{ll'}, \mathbf{p}_{l'l}$ are thus defined up to $D\,N\,(N-1)/2$ degrees of freedom ($\lambda$ and $\mathbf{w}_{ll'}^{\perp} \in \mathbb{R}^D$ subject to an orthogonality constraint, for each category pair).

Furthermore, from a geometrical point of view, it is coherent to require that $l_{\mathbf{p}_{ll'}} = l$, in words that each prototype belongs to a polytope $\Omega_l$ corresponding to the label $l$, i.e., that $\forall l, l', l'' l \neq l', l \neq l'', \mathbf{w}_{ll''}^T\,\mathbf{p}_{ll'} + w_{ll''}^0 > 0$. This corresponds to a linear programming problem with $D\,N$ degrees of freedom and $(N\,(N-1)/2)^2$ inequalities, thus with solutions in the general case as soon as $D > N^3/4 + O(N^2)$, i.e., as soon as the number of features is high enough with respect to the number of categories.

Is it possible to reduce the number of prototypes, i.e., that $\mathbf{p}_{ll'} \stackrel{?}{=} \mathbf{p}_{ll''}$ for some of the $N\,(N-1)$ prototypes ? This generates $N$ additional linear constraints for each prototype merge, and the non trivial fact that prototype pairs are to live into the same connected component of a given $\Omega_l$ has to be taken into account. A simple count of the number of degrees of freedom shows that the number of constraints is in the general case twice the number of possible adjustment. Prototype merge is thus possible, but not completely.

The softmax decision rules is thus equivalent to a nearest-neighbor algorithm considering $O(N^2)$ prototypes.

## APPENDIX B: HYPER PARAMETERS ANALYSIS

As far as significance and interpretability of the results is concerned a key point is the influence and adjustment of the algorithm hyper-parameters. We have already discussed those for which it was worth studying specifically their influence, but let us now briefly review exhaustively all of them.

Experiments are performed in Python with help of basic scientific libraries, especially the machine learning library scikit-learn (Pedregosa et al., 2011), from which comes implementations for k-means and cross-entropy multinomial (softmax) regression.

Regarding the k-means algorithm and its k-means++ initialization heuristic, we have to consider:
- The number $J$ of clusters, studied in this section.
- The number $R$ of random draws of initial conditions of the expectation-minimization algorithm.
- The maximal number $K$ of iteration of the expectation-minimization algorithm.
- The tolerance $E$ on the criterion variation in order to detect the convergence.
- The extended criterion hyper-parameter $\beta$ introduced in the previous section.
- A choice between two algorithm variants which mainly differ in computational efficiency, the faster one being chosen.

Here given a data set we easily compute the maximal and minimal distances between clusters:

$$M \stackrel{\mathrm{def}}{=} \max_{ii'} |\mathbf{x}_i - \mathbf{x}_{i'}|^2 \text{ and } m \stackrel{\mathrm{def}}{=} \min_{ii'} |\mathbf{x}_i - \mathbf{x}_{i'}|^2,$$

allowing us to adjust of fix these hyper-parameters.

We have observed that the number $R$ of random draws is not significant as soon as sufficient (typically $R > 10$). The maximal number of iterations $K$ has not to be bounded because the algorithm always converges. The tolerance $E$ is easy to infer from the data, because as soon as the expectation step of the k-means algorithm does not "redistribute" samples between clusters the algorithm is expected to converge in one step. This occurs as soon as the criterion variation magnitude is lower than the minimal distance between samples, i.e. as soon as $E \ll m$, say 10 times lower.

The number $J$ of clusters is to be adjusted as studied in this section, but we know in which bounds, since we can easily set as minimal number the number of categories (i.e., considering one cluster by category), and set as maximal number the number of learning samples (i.e., falling back to a nearest-neighbor algorithm).

Finally, through $\beta$ is a parameter to be observed and adjusted as studied in this section, we also know in which bounds, since $\beta = 0$ corresponds to the not taking a-priori information into account and $\beta = M$ to hard-wire prototypes on categories, as analyzed in Appendix A.4.

Regarding the second step of the method — the cross-entropy minimization — we have to consider similarly:
- The choice of the minimization solver.
- The maximal number $K$ of iterations of the minimization algorithm.
- The tolerance $E$ on the criterion variation in order to detect the convergence.
- The choice of the $\mathcal{L}^D, D \in \{1, 2\}$ regularization.
- The regularization balance weight $C$.

A softmax function being considered here, we can again calculate the output variation under which the algorithm convergence is negligible. The classification decision does not vary for variations of the output below $c = \min_{jj'} |c_j = c_{j'}|$ as being a comparison between two outputs, while $c_j \in [0, 1]$.

For L2 penalty, L-BFGS solver was chosen for its faster convergence (less iterations). The algorithm always converged allowing us not to consider $K$ as a significant value, at a tolerance $E = 10^{-4}$. Given some restrictions by the library, only one solver option was available for L1 penalties (SAGA solver). Tolerance was relaxed for SAGA solver so as to achieve convergence within the same $K$ iterations ($E = 10^{-1}$). The initial point for both solvers is always taken at zero, thus no hyper-parameter or heuristic is to be considered.

Beyond these parameters, the more significant parameters $C$ and $D$ have been studied in the text.

In addition to these two sets of parameters we have discussed the $\alpha \in [0, 1]$ shortcut gain allowing us to better understand the performances ans limit of our method.

All together, the literature knowledge, simple rule of thumbs on the data values, the concrete understanding of the proposed algorithms and specific numerical studies of more critical parameters allows us to both propose a reproducible piece of experimental results and a method than can be reused without any opaque or application dependent hyper-parameters adjustment, that are not done by the hyper-parameter adjustment layer or the proposed method.

## REFERENCES

Pedregosa, F., Varoquaux, G., Gramfort, A., Michel, V., Thirion, B., Grisel, O., et al. (2011). Scikit-learn: Machine learning in Python. *Journal of Machine Learning Research* 12, 2825–2830
